# Condensate nanovaccine adjuvants augment CD8^+^ T-Cell-dependent antitumor immunity through mtDNA leakage-triggered cGAS-STING axis activation

**DOI:** 10.1038/s41392-025-02447-w

**Published:** 2025-10-21

**Authors:** Yu Tang, Zhiyuan Luo, Zhanni Ma, Lingling Han, Yurong Zhou, Tianci Liang, Kangsen Yang, Lei Zhao, Xiaoyuan Chen, Pengfei Zhang

**Affiliations:** 1 Institute of Molecular Immunology, School of Laboratory Medicine and Biotechnology, Southern Medical University, Guangzhou, China; 2 Innovation Research Institute of Traditional Chinese Medicine, Shanghai University of Traditional Chinese Medicine, Shanghai, China; 3 Department of Radiology, Zhongnan Hospital of Wuhan University, Wuhan, Hubei Province China; 4 Department of Orthopedics, Air Force Hospital of Eastern Theater of PLA, Nanjing, China; 5 Departments of Diagnostic Radiology, Surgery, Chemical and Biomolecular Engineering, Biomedical Engineering, Clinical Imaging Research Centre, Pharmacy and Pharmaceutical Sciences, and Nanomedicine Translational Research Program, Yong Loo Lin School of Medicine and Faculty of Engineering, National University of Singapore, Singapore, Singapore; 6 Institute of Molecular and Cell Biology, Agency for Science, Technology and Research (A*STAR), Singapore, Singapore; 7 Theranostics Center of Excellence (TCE), Yong Loo Lin School of Medicine, National University of Singapore, Singapore, Singapore

**Keywords:** Nanobiotechnology, Tumour immunology, Drug development, Drug delivery

## Abstract

The variety and functionality of current clinical vaccine adjuvants remain limited. Conventional aluminum-based adjuvants predominantly induce Th2-biased humoral immunity but exhibit a limited capacity to elicit Th1-mediated cellular immune responses, particularly tumor antigen-specific cytotoxic CD8^+^ T lymphocytes (CTLs), which are essential for effective cancer vaccine performance. Inspired by natural biomolecular condensates, we developed a versatile noncovalent protein self-assembly strategy distinct from traditional approaches requiring structural domain modifications or bifunctional crosslinkers. Our methodology employs amphiphilic molecules (sodium myristate/SMA and sodium dodecyl thiolate/SDT) as molecular bridges to mediate protein‒protein interactions through hydrophobic forces and disulfide bond formation. This process generates nanoscale protein condensate (PCD) vaccines with exceptional stability. As a novel adjuvant system, these synthetic condensates significantly enhance antigen cross-presentation by optimizing key parameters: antigen loading capacity, lymph node targeting, cytosolic delivery, and lysosomal escape. Consequently, they induce robust antigen-specific CTL responses and humoral immunity, demonstrating potent antitumor efficacy. Importantly, we found that the synthetic protein condensate (PCD) alone can act as a nanoadjuvant. By increasing mitochondrial membrane permeability, PCD induces mitochondrial DNA leakage into the cytosol, activating the cGAS‒STING pathway and promoting DC maturation. This safe and scalable platform eliminates the need for complex covalent modifications or genetic engineering, and it facilitates the design of diverse modular antigens, including neoantigens and viral antigens. Given its straightforward manufacturing process and superior immunogenicity, this synthetic PCD vaccine adjuvant has significant potential for clinical application and translation.

## Introduction

Vaccine adjuvants serve as critical immunomodulators that amplify the magnitude and quality of the immune response through various mechanisms, including antigen-presenting cell (APC) activation, controlled antigen release, and enhanced delivery efficiency.^1–5^ Aluminum-based adjuvants (e.g., aluminum hydroxide/phosphate) dominate clinical practice because their established safety profile and ability to produce antibodies are particularly suitable for prophylactic vaccines.^6–8^ However, their limited capacity to induce Th1-type cellular immunity restricts their therapeutic applications.^9–11^ Emerging adjuvants (MF59,^12^ AS01,^13^ and CpG oligonucleotides^14^) address this limitation by stimulating CD8^+^ T-cell responses critical for pathogen/tumor clearance^15,16^ but face challenges in terms of material availability, manufacturing complexity, and safety evaluation.^17–19^ These constraints underscore the need for next-generation adjuvants that combine efficacy, safety, and scalable production.

Current research on cancer vaccines is characterized by two major trends.^17–22^ On one hand, the development of neoantigen vaccines is driving the rapid expansion of personalized cancer immunotherapy. On the other hand, vaccine platforms are increasingly being combined with immune checkpoint inhibitors, cellular therapies, or physicotherapeutics to create new treatment regimens. Despite these advances, the clinical translation of cancer vaccines still faces significant challenges: (1) the identification and validation of neoantigens is a complex and time-consuming process, and many candidate neoantigens display insufficient immunogenicity in clinical or preclinical settings, which constrains antigen selection; (2) antigen delivery systems often exhibit suboptimal cross-presentation efficiency and limited in vivo stability, resulting in inadequate cytotoxic T lymphocyte (CTL) responses; and (3) immunosuppressive signals within the tumor microenvironment—such as infiltration by regulatory T (Treg) cells and secretion of immunosuppressive factors—substantially attenuate vaccine efficacy. Integrative approaches that combine biology with chemistry and materials science, in particular the introduction of novel nanovaccines, may help to overcome these bottlenecks in the cancer vaccine field. Nanovaccines represent a frontier in cancer immunotherapy through the use of antigen nanocarriers (liposomes, polymeric/metal-organic nanoparticles) that increase antigen delivery, APC uptake, and lymph node targeting.^20–23^ Advanced platforms integrate checkpoint inhibitors to amplify antitumor immunity.^24–27^ Nevertheless, the clinical translation of nanoadjuvants remains hindered by their suboptimal antigen loading (<10%),^28–30^ manufacturing complexity,^31–33^ and nanomaterial toxicity.^34^ Current innovations focus on biomimetic carriers,^35^ microfluidic optimization,^36^ and surface engineering;^37^ however, balancing safety with delivery efficiency and production simplicity remains a critical challenge. Besides, efficient intracellular protein delivery technologies are critical for the development of therapeutics that target intracellular proteins, and are of particular importance in the field of protein-based nanomedicines. A major challenge is achieving effective endosomal escape of protein cargoes after cellular uptake; endosomal escape is a prerequisite for delivered proteins to exert their biological functions. Therefore, a simple, readily scalable delivery platform that enables efficient intracellular transport while preserving protein bioactivity is still an ideal goal.

Recent studies have demonstrated that biomolecular condensates, which form via liquid‒liquid phase separation and noncovalently co-assembled of proteins, nucleic acids, and other biomacromolecules, can compartmentalize cellular biochemical reactions, thereby regulating cellular processes.^38–42^ These condensates have been implicated in neurodegenerative diseases and cancer, suggesting promising targets for therapeutic intervention.^43,44^ With ongoing research of the mechanisms underlying biomolecular condensates, the application of synthetically engineered condensates for immunomodulation and disease therapy holds great promise as an emerging biomedical technology. Compared with conventional protein engineering methods (e.g., genetic fusion and covalent conjugation), which may damage native protein structures,^45–51^ artificial biomolecular condensates could serve as alternative approaches.

**Fig. 1:**
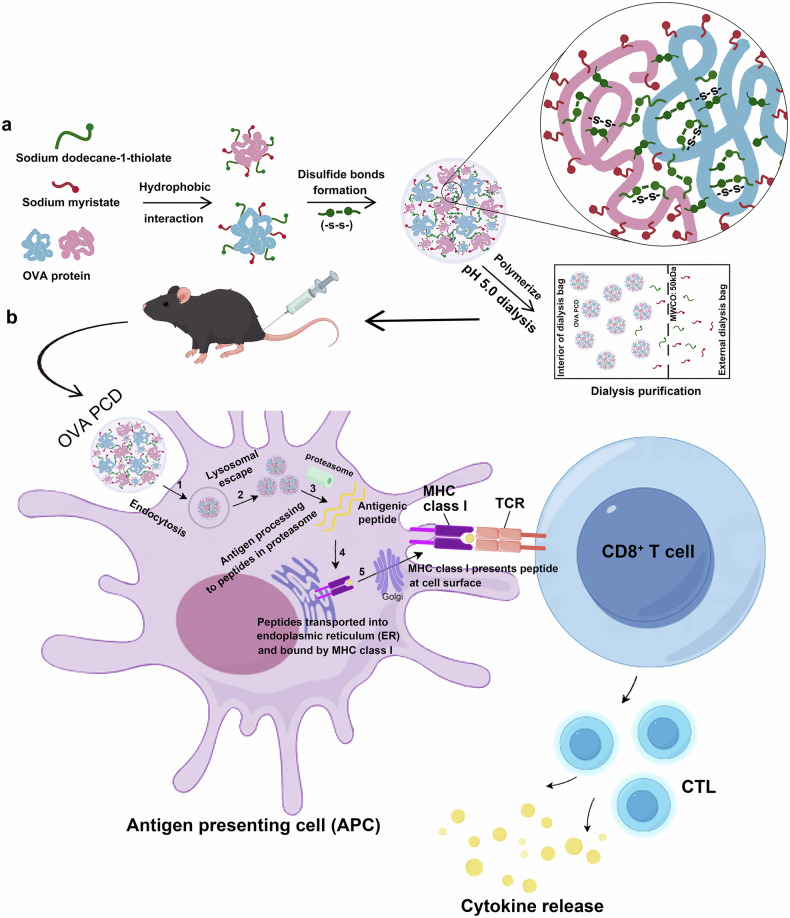
Schematic illustration of the mechanism underlying the PCD-mediated antitumor CD8^+^ T-cell immune response. **a** Sodium myristate (SMA, red amphiphilic molecules) and sodium dodecane-1-thiolate (SDT, green amphiphilic molecules) are adsorbed onto the surface of proteins through hydrophobic interactions. Subsequently, SDT molecules on the OVA surface promote the formation of disulfide bonds (-S-S-) between thiol groups (-SH) via oxidation reactions, allowing SDT to act as a molecular bridge that connects OVA-surfactant complexes. Ultimately, under mildly acidic conditions (pH = 5.0), the partial protonation of these amphiphilic molecules transforms them from soluble substances into hydrophobic molecules, further enhancing the hydrophobic interactions between the protein and molecular bridges and promoting the self-assembly of protein-SMA-SDT intermediates into protein condensates. By precisely adjusting the fatty acid-mediated hydrophobic interactions (by modulating the acidic pH of the buffer) and the formation of disulfide bonds, the size, morphology, and stability of OVA protein condensates (OVA PCD) can be well controlled. **b** After subcutaneous injection, OVA PCD activates CD8^+^ T cells through antigen cross-presentation. First, synthetic PCD is endocytosed by DC cells (1) and then transported via the endosomal pathway, with a portion of OVA PCD escaping from the endosome (2). This protein is subsequently degraded by the proteasome in the cytosol (3), and the resulting peptide fragments are transported to the endoplasmic reticulum (ER), where they are loaded onto MHC-I molecules (4). Finally, the epitope peptides are presented on the cell membrane to antigen-specific CD8^+^ T cells (5), which, upon stimulation, proliferate and differentiate into CD8^+^ cytotoxic T lymphocytes (CTLs) to exert potent tumor-killing effects. The figure was created via resources from FigDraw (www.figdraw.com)

Inspired by natural biomolecular condensates, we innovatively adopted a noncovalent protein coassembly strategy to develop a new type of synthetic protein-biomolecular condensate (PCD) vaccine. DC cells can be activated through the induction of mtDNA leakage via PCD to trigger the cGAS‒STING axis, thereby enhancing CD8⁺ T-cell-dependent antitumor immunity. Briefly, amphiphilic molecules (sodium myristate/SMA and sodium dodecane-1-thiolate/SDT) were mixed with protein ligands, enabling their adsorption onto the protein surface. Under oxygen and acidic buffer conditions, these amphiphilic molecules subsequently act as “molecular bridges” to drive the self-assembly and aggregation of various protein ligands through hydrophobic interactions (protonation of SMA converts it from hydrophilic to hydrophobic), and disulfide bonds form between protein ligands (oxidation enables SDT molecules to form stable disulfide bonds while rendering SDT hydrophobic). This process ultimately yields synthetic protein-biomolecular condensates (PCDs) (Fig. 1a). Sodium myristate (SMA), a natural fatty acid derivative, was prioritized as the primary self-assembly component because of its favorable metabolic clearance and biosafety profile in vivo. Under acidic conditions (pH 5.0), partial protonation of SMA enhances its hydrophobicity, driving the aggregation and self-assembly of protein-SMA complexes. Concurrently, sodium dodecanethiolate (SDT) undergoes intermolecular oxidation to form disulfide bonds in PCDs, significantly improving nanoparticle stability (Supplementary Fig. 1). As a novel adjuvant system, this condensate vaccine improved the loading efficiency of protein antigens in carriers via a protein‒ligand coassembly strategy. Furthermore, this adjuvant system enhances lymph node targeting, cytosolic delivery, and lysosomal escape of antigens to amplify cross-presentation, significantly eliciting potent cytotoxic T lymphocyte (CTL) responses and tumor regression (Fig. 1b). The modular design accommodates diverse antigens (neoantigens/viral antigens) without complex modifications, offering manufacturing simplicity and therapeutic versatility against malignancies, thus highlighting the significant clinical potential of condensate vaccines.

## Result

### OVA PCD formulation and characterization

**Fig. 2:**
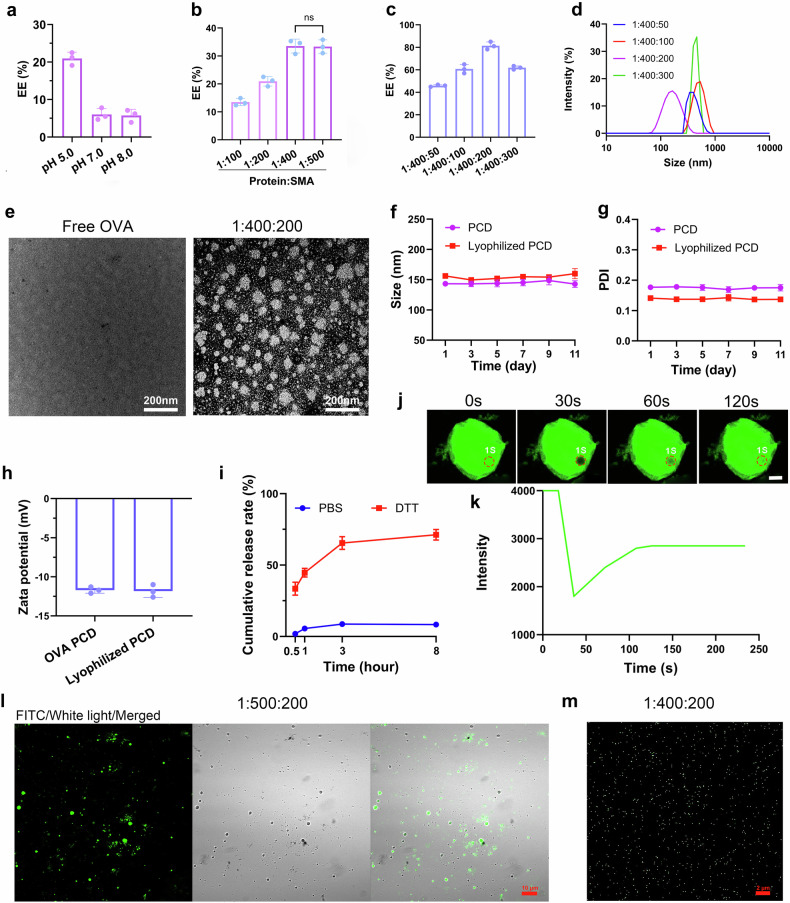
Optimization of preparation conditions and characterization of OVA PCD. **a** OVA was mixed with SMA at a molar ratio of 1:200. The protein encapsulation efficiency (EE%) of the primary complex (OVA-SMA) under different pH conditions was evaluated (*n* = 3). **b** Comparison of protein encapsulation efficiency (EE%) for OVA-SMA complexes prepared at different molar ratios under pH 5.0 dialysis (*n* = 3). **c** Under dialysis conditions at pH 5.0, the protein encapsulation efficiency (EE%) of OVA PCD was evaluated by mixing the primary OVA-SMA complex with different ratios of sodium dodecyl thiolate (SDT) (*n* = 3). **d** DLS analysis of OVA PCD particles after mixing at different OVA:SMA (sodium myristate):SDT (sodium dodecanethiolate) molar ratios. **e** TEM images of OVA PCD at a ratio of 1:400:200 (OVA:SMA:SDT) and free OVA. Scale bar: 200 nm. Evaluation of the particle size stability (*n* = 3, **f**) and corresponding PDI values (*n* = 3, **g**) of OVA PCD over 11 days before and after lyophilization. **h** Changes in the zeta potential of OVA PCD before and after lyophilization (*n* = 3). **i** Cumulative release profile of OVA PCD under in vitro reducing conditions (*n* = 3). Fluorescence recovery after photobleaching (FRAP) assay of the material after dialysis of OVA with sodium myristate (SMA) at a 1:400 molar ratio, as measured by confocal microscopy (**j**), with corresponding quantitative analysis of fluorescence intensity (**k**). The red dashed circles indicate the photobleached regions. Scale: 5 μm. **l**, **m** Confocal fluorescence images of FITC-labeled artificial condensates. Micron-sized particles formed at a 1:500:200 ratio (**l**); scale bar: 10 µm. Nanosized particles formed at a 1:400:200 ratio (**m**); scale bar: 2 µm. All data (except for **d**, **k**) are presented as the mean ± s.d. (*n* = 3) from three independent experiments

To optimize the formulation of the amphiphilic molecules sodium myristate (SMA) and sodium dodecyl thiosulfate (SDT) for promoting ovalbumin (OVA) self-assembly into nanocondensates, OVA was initially complexed with SMA (1:200 molar ratio) and dialyzed against buffers at various pH values, forming primary complexes (P-SMA). Protein encapsulation efficiency was subsequently quantified. As indicated in Fig. 2a, the encapsulation efficiency was markedly enhanced under weakly acidic conditions (pH 5.0) relative to neutral and alkaline environments. This phenomenon is attributed to the protonation of partial SMA under acidic pH (pH 5.0), which shifts its amphiphilic balance toward hydrophobicity. This transition promotes SMA adsorption onto the protein surface and subsequent aggregation, facilitating efficient protein encapsulation.

Having identified pH 5.0 as the optimal dialysis condition, we observed that the encapsulation efficiency progressively increased with increasing SMA:protein molar ratios (Fig. 2b), achieving a maximum efficiency at 400:1. Further formulation optimization demonstrated that an OVA:SMA:SDT molar ratio of 1:400:200 yielded ideal nanoparticle characteristics, including optimal encapsulation efficiency and size distribution (Fig. 2c, d, Supplementary Figs. 1, 2). TEM analysis also confirmed that OVA PCDs formed at this ratio resulted in uniform spherical nanostructures (50–100 nm diameter; Fig. 2e), in contrast with the free OVA samples which exhibited negligible nanoparticle formation. Additionally, OVA PCDs prepared at a 1:400:200 ratio maintained a consistent particle size and low polydispersity (PDI < 0.2) over an 11-day period (Fig. 2f, g) while exhibiting a net negative surface charge (Fig. 2h). In vitro release experiments (Supplementary Fig. 3) demonstrated that, in a simulated physiological environment (PBS, pH 7.4), the cumulative release rate of synthetic OVA-PCD was less than 10% over 144 h. This finding indicates good structural stability and sustained release properties, which may effectively protect the antigen from degradation and extend its action time in vivo. Therefore, we further added DTT (20 mM) to the OVA PCD solution to simulate the reducing environment of the cytoplasm. The results revealed that the cumulative release from synthetic PCD reached approximately 71% within 8 h of DTT treatment, whereas it was only approximately 8.5% in PBS (Fig. 2i). This finding indicates that DTT cleaves disulfide bonds in synthetic PCD, destabilizes the particles, and triggers disassembly, releasing the cargo protein. This ensures that PCD can control intracellular drug release.

Next, to verify whether the synthetic protein nanoclusters have the droplet-like properties of natural biomolecular condensates, we conducted fluorescence recovery assays after photobleaching (FRAPs) on primary complexes (Protein-SMA) formed by FITC-labeled OVA and SMA (1:400) via a fluorescence confocal microscope. After photobleaching a region, the fluorescence recovered starting at 30 s, reaching 70% of the initial intensity by 120 s (Fig. 2j, k). Moreover, at a 1:500:200 assembly ratio, micrometer-sized droplet-like particles formed (Fig. 2l); at a 1:400:200 ratio, nanosized particles formed (Fig. 2m). This finding shows that PCD is in a liquid-like state and that the size can be controlled by adjusting the SDT ratio. Finally, we also coassembled the SARS-CoV-2 S glycoprotein and influenza HA antigen into a PCD vaccine. Nanoflow cytometry revealed that approximately 93.7% of the particles could carry both antigens (Supplementary Fig. 4), confirming that our synthetic PCD technology has versatility and modularity.

### Lysosomal escape properties and in vitro biocompatibility of OVA PCD

**Fig. 3:**
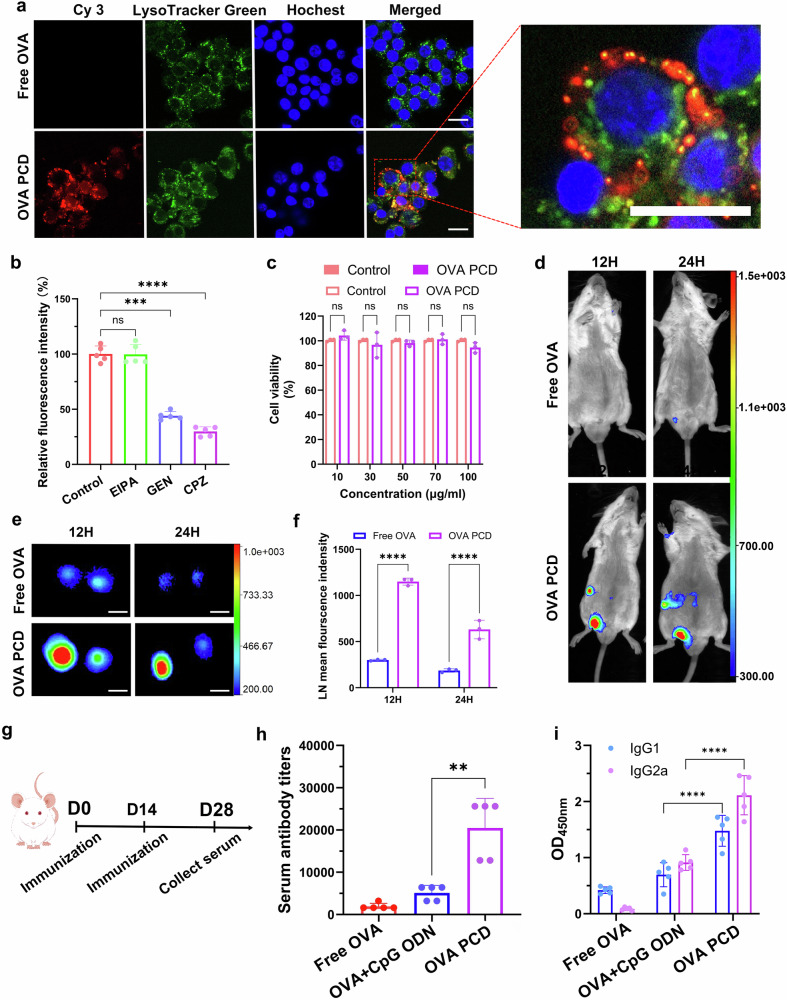
Lysosomal escape, biocompatibility, lymph node targeting, and enhanced humoral immune response to OVA PCD. **a** OVA PCD representative fluorescence micrographs of cytosolic delivery via lysosomal escape. Confocal microscopy images revealed that after 12 h of treatment with Cy3-labeled OVA PCD (2.5 μg/mL, red) or free OVA protein (2.5 μg/mL, control) in RAW 264.7 cells, the nuclei were stained with Hoechst dye (blue), and the lysosomes were stained with LysoTracker dye (green). OVA PCD did not colocalize with the lysosomes, indicating successful escape from the lysosomes into the cytoplasm. Scale bar: 20 μm. **b** Analysis of the cellular uptake pathways of OVA PCD. Ethamilamiline (EIPA), genistein (GEN), and chlorpromazine (CPZ) were used to inhibit macropinocytosis, caveolin-mediated endocytosis, and clathrin-mediated endocytosis, respectively (*n* = 5). **c** Cytotoxicity of OVA PCD at different concentrations (10, 30, 50, 70, and 100 μg/mL) was assessed via a CCK-8 assay after coincubation with 293 T cells for 24 h (n = 3). **d**–**f** C57BL/6 mice were subcutaneously injected at the tail base with 50 µg of Cy5.5-labeled OVA-PCD (equivalent to the same amount of free OVA, *n* = 3). Representative images of in vivo fluorescence signals in the draining inguinal lymph nodes (LNs) were acquired via the FX Pro small animal imaging system at 12 and 24 h post-injection (**d**). LNs were harvested at the corresponding time points for ex vivo fluorescence signal acquisition (**e**), scale bar: 0.5 cm, and considering the leftward bias in the distribution of the fluorescence signal, a quantitative analysis of the relative fluorescence intensity was performed on the left lymph nodes (**f**). **g** Timeline of the in vivo humoral immune study of OVA PCD. **h** Total antibody titers in the serum of BALB/c mice after immunization with different formulations twice (immunized every two weeks, *n* = 5). **i** Detection of different IgG subtype levels in the serum of BALB/c mice after immunization with different formulations twice (immunized every two weeks) following serum dilution (*n* = 5). All data are expressed as mean ± s.d. from two independent experiments. Unpaired two-tailed Student’s *t* tests were used for (**c**, **f**). One-way ANOVA with Tukey’s post hoc test was employed for (**b**, **h**, **i**) Significance levels are indicated as **p < 0.01, ***p < 0.001, ****p < 0.0001, and ns (not significant)

Next, we investigated the intracellular delivery efficiency of synthetic OVA PCD. OVA PCD was labeled with Cy3-NHS. The labeled OVA PCD was coincubated with RAW 264.7 cells for 12 h, after which the lysosomes were stained with the green fluorescent probe LysoTracker Green, and the nuclei were stained with Hoechst 33342. Confocal laser scanning microscopy (CLSM) colocalization analysis revealed that the red fluorescence (Cy3-NHS-labeled OVA PCD) was almost completely separated from the green fluorescence (LysoTracker Green-labeled lysosomes). This finding indicates that OVA PCD was successfully internalized by the cells and could escape from the lysosomes into the cytoplasm (Fig. 3a), thereby achieving the cytoplasmic delivery of antigens.

To further validate the lysosomal escape mechanism of this synthesized PCD, we conducted additional coincubation experiments with Cy3-labeled PCD and DC2.4 cells. By examining lysosomal colocalization at 12, 24, and 48 h, we observed the dynamic intracellular trafficking of PCD. The results (Supplementary Figs. 5 and 6) demonstrated that PCD without Tween 80 was internalized into lysosomes by DC2.4 cells at 24 h and initiated lysosomal escape by 48 h. Moreover, the addition of Tween 80 significantly accelerated this process: synthetic PCD containing Tween 80 was internalized into lysosomes as early as 12 h and almost completely translocated into the cytoplasm by 48 h. Tween 80 in the PCD formulation significantly enhanced nanoparticle cellular internalization. Therefore, given that endocytosis is likely the primary route of PCD internalization, we used various inhibitors—EIPA (a macropinocytosis inhibitor), CPZ (a clathrin-mediated endocytosis inhibitor), and GEN (a caveolae-mediated endocytosis inhibitor)—to monitor OVA PCD uptake. The results revealed that PCD primarily entered cells through clathrin- and caveolae-mediated endocytosis (Fig. 3b), confirming the endocytosis-based mechanism of PCD. Although the mechanism of lysosomal escape by PCD is not fully understood, we hypothesize that sodium myristate and Tween 80, which act as amphiphilic surfactants, integrate into lysosomal membranes to destabilize their integrity, thereby facilitating PCD nanoparticle escape into the cytosol.

Finally, the biocompatibility of OVA PCD was further evaluated at the cellular level. Different concentrations of PCD were coincubated with 293 T cells in a 96-well plate for 24 h, and cell viability was assessed via the CCK-8 assay. The results showed that OVA PCD did not exhibit significant cytotoxicity across the tested concentration range (Fig. 3c). These findings suggest that the synthetic PCD system constructed in this study not only achieves efficient cytoplasmic delivery of antigens but also meets the safety requirements of biomedical materials.

### Lymph node-targeting delivery properties and enhanced humoral immune response of PCD

To systematically evaluate the in vivo delivery characteristics of OVA PCD, we labeled both OVA PCD (Cy5.5-OVA PCD) and free OVA (Cy5.5-OVA) with the near-infrared fluorescent probe Cy5.5. Their biodistribution was then quantified at 12 and 24 h after subcutaneous injection (s.c.) at the tail base of the mice via small animal in vivo imaging technology. The results (Fig. 3d–f) revealed that at the 12-h time point, both formulations produced significant fluorescence signals in the left lymph nodes (LNs). However, the fluorescence intensity of Cy5.5-OVA PCD in the left inguinal lymph nodes was significantly greater than that in the free OVA group. Further relative fluorescence quantification of the left lymph nodes confirmed that the lymph node (LN)-targeting efficiency of PCD was approximately 3.8 times greater than that of free OVA (Fig. 3f). Moreover, significant signal intensity was maintained at 24 h, whereas the lymph node fluorescence signal in the free OVA group nearly disappeared, indicating that synthetic PCD has excellent specific delivery capability to lymphatic system. Furthermore, OVA PCD was subcutaneously administered to another batch of mice, and the lymph nodes were collected for immunohistochemical staining analysis 24 h later. The results demonstrated that OVA efficiently trafficked to the lymph nodes (Supplementary Fig. 7), further confirming its efficient lymph node-targeting ability. However, OVA PCD is distributed unevenly in the lymph nodes and mainly accumulates at the periphery before slowly moving toward the center. This may be due to the size of the nanoparticles, which passively enter the lymph nodes via lymphatic drainage, become trapped, and are retained long-term.

Thus, after injecting specific formulations into mice and analyzing serum antibody titers and IgG subtypes (Fig. 3g), synthetic OVA PCD immunization resulted in significantly higher total antibody titers than the traditional CpG ODN adjuvant (OVA + CpG ODN) (Fig. 3h). IgG subtype analysis revealed increased levels of both IgG1 and IgG2a, with IgG2a levels in the OVA PCD group being twice as high as those in the OVA + CpG ODN group (Fig. 3i). These results indicate that OVA PCD enhances antigen immunogenicity by stimulating B-cell activation through antigen polymerization on PCD and simultaneously activating Th1 and Th2 immune responses (Fig. 3i).

### In vivo antitumor effects and immunological mechanisms of OVA PCD

**Fig. 4:**
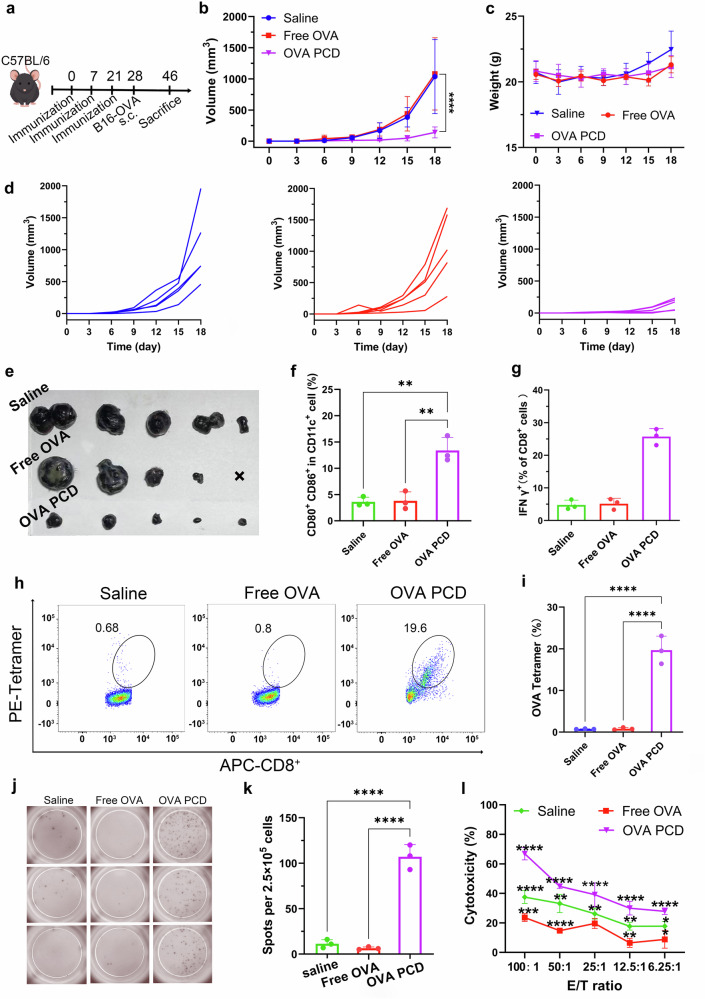
Evaluation of the in vivo antitumor efficacy and immunological mechanisms of OVA PCD. **a** Schematic timeline of vaccine and B16-OVA tumor cell inoculation in the C57BL/6 mouse model. The mice were immunized three times via subcutaneous injection at the tail base with OVA PCD, free OVA, or saline. On the seventh day after the final immunization, 5 × 10^5^ B16-OVA tumor cells were subcutaneously inoculated into the right thighs of the mice. **b** Tumor growth curves within 18 days after the subcutaneous inoculation of B16-OVA tumor cells. Compared with that in the free OVA or saline group, tumor growth in the OVA PCD group was significantly inhibited (*n* = 5). **c** Body weight change curves after tumor inoculation. The OVA PCD group showed no significant change in body weight, indicating that there was no apparent systemic toxicity (*n* = 4–5). **d** Individual tumor growth curves for each group after subcutaneous inoculation of B16-OVA cells. **e** Tumor images from day 18 posttumor inoculation. “x” indicates mice that died on day 18 posttumor inoculation (experimental endpoint). The tumor volume data of this deceased mouse from the statistical analysis for day 18 are included in Fig. 4b. The data in **b**, **c** are presented as mean ± s.d. from two independent experiments. Groups were compared via one-way ANOVA with Tukey’s post hoc test. Flow cytometry analysis of the DC maturation rate (CD80^+^ CD86^+^ CD11c^+^) (**f**) and the percentage of IFN-γ^+^ CD8^+^ T cells (**g**) in the spleens of mice subjected to different treatments (*n* = 3). Representative flow cytometry results (**h**) and quantification (**i**) of OVA antigen peptide-specific CD8^+^ T cells after ex vivo restimulation with the SIINFEKL peptide (8 μg/mL) (*n* = 3). Representative images (**j**) and statistical analysis (**k**) of IFN-γ spot-forming cells in splenocytes after ex vivo restimulation with the SIINFEKL peptide (8 μg/mL) via the ELISPOT assay (*n* = 3). **l** Evaluation of the cytotoxic effect of effector splenocytes (E) on target B16-OVA-GFP cells (T) after coincubation for 24 h at specified ratios, assessing the in vitro tumor cell-targeting cytotoxicity of effector T cells (*n* = 3). Statistical analysis data in (**f–l**) are presented as mean ± s.d. from three independent experiments. Groups were compared via one-way ANOVA with Tukey’s post hoc test. Significance levels are indicated as **p < 0.01, ***p < 0.001, ****p < 0.0001, and ns (not significant)

The in vivo antitumor efficacy of OVA PCD was further investigated by immunizing mice three times consecutively with OVA PCD. On the seventh day after the final immunization, 5 × 10^5^ B16-OVA melanoma cells were subcutaneously injected into the right thigh, and tumor growth was monitored over 18 days (Fig. 4a). The experimental results revealed striking differences in tumor progression among the treatment groups. Both the saline and free OVA control groups exhibited exponential tumor growth, reaching mean volumes of (1.04 ± 0.52) × 10^3^ mm^3^ and (1.09 ± 0.51) × 10^3^ mm^3^, respectively, by day 18 postinoculation, with no statistically significant difference between them (Fig. 4b). In contrast, compared with control treatment, OVA PCD profoundly suppressed tumor progression, limiting the final tumor volume to merely (0.12 ± 0.08) × 10^3^ mm^3^—an 88% reduction (Fig. 4b). Critically, no significant body weight fluctuations were observed in the OVA PCD group throughout the study (Fig. 4c), confirming the favorable safety profile of the formulation and the absence of systemic toxicity. Terminal endpoint analyses further corroborated these findings: gross anatomical examination and tumor resection revealed that the OVA PCD-treated mice presented significantly smaller tumors (Fig. 4d, e) than did the saline-treated and free OVA-treated mice. These findings confirmed that OVA PCD effectively delayed the progression of solid tumors.

To elucidate the underlying mechanisms, splenic lymphocytes from each group were isolated at the experimental endpoint (day 18 posttumor inoculation) for multidimensional immunophenotypic analysis. Flow cytometry revealed that the percentage of CD11c^+^ CD80^+^ CD86^+^ mature dendritic cells in the OVA PCD group reached 13.4 ± 2.0%, which was significantly greater than that in the free OVA group (3.81 ± 1.39%) and the saline group (3.62 ± 0.72%) (Fig. 4f, Supplementary Fig. 8). These findings suggest that OVA PCD enhances antigen cross-presentation, promoting dendritic cell maturation and costimulatory molecule expression. In the spleens of the mice in the PCD group, the percentage of IFN-γ^+^ CD8^+^ T cells was 25.7 ± 2.0% of the total CD8^+^ T cells, and the percentage of OVA tetramer^+^ CD8^+^ T cells was 19.7 ± 2.7% of the total CD8^+^ T cells, both of which were significantly greater than those in the saline group (4.7 ± 1.2%, 0.73 ± 0.05%) and the free OVA group (5.1 ± 1.3%, 0.80 ± 0.23%) (Fig. 4g–i, Supplementary Figs. 9-10). These findings indicate that OVA PCD induces antigen-specific cytotoxic T lymphocyte (CTL) responses. Additionally, IFN-γ ELISPOT assays further confirmed that the number of spot-forming cells (SFCs) in the OVA PCD group was 107 ± 11 spots, which was significantly greater than that in the free OVA group (6 ± 1 spots) and the saline group (11 ± 4 spots) (Fig. 4j‒k), revealing the capacity of OVA PCD treatment to induce clonal proliferation of antigen-specific T cells. To assess the functional activity of effector T cells after immunotherapy, splenocytes from each group were cocultured with B16-OVA-GFP tumor cells at graded E/T ratios (6.25:1 to 100:1) for 24 h in vitro, followed by evaluation of effector T-cell cytotoxicity. As shown in Fig. 4l, at the highest E/T ratio (100:1), the tumor cell killing efficiency of the effector T cells in the OVA PCD group reached 66.8 ± 3.3%, which was significantly greater than that in the free OVA group (23.6 ± 2.1%) and the saline group (37.4 ± 3.5%) and maintained approximately 30% cytotoxicity even at the lowest ratio (6.25:1). Furthermore, fluorescence microscopy (Supplementary Fig. 11) revealed significant apoptosis in tumor cells from the OVA PCD-treated group (reduced GFP fluorescence), which was consistent with the results shown in Fig. 4l. In addition, we confirmed the absence of adjuvant-like contaminants by quantifying endotoxin levels in OVA (Supplementary Fig. 12), ensuring that the observed immunostimulatory effects were attributable solely to the PCD nanoparticles themselves. In summary, OVA PCD significantly enhances antitumor immune responses by increasing antigen presentation, inducing CTL responses, and promoting antigen-specific T-cell clonal proliferation, demonstrating promising CD8^+^ T-cell-mediated antitumor therapeutic effects.

### Validation of universality and toxicological evaluation

**Fig. 5:**
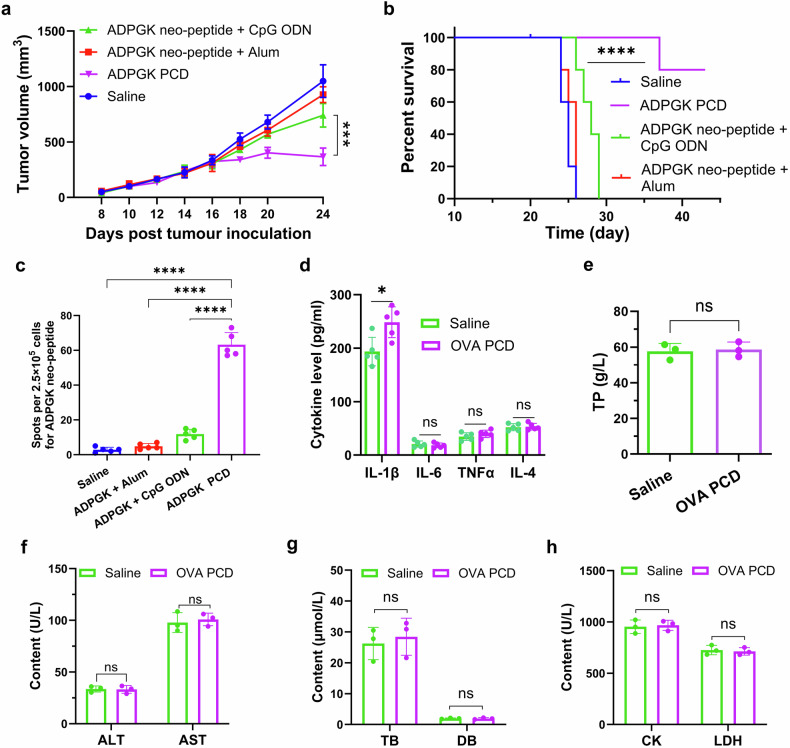
In vivo antitumor activity of ADPGK PCD. **a**, **b** C57BL/6 mice were subcutaneously inoculated with 2 × 10^5^ MC-38 tumor cells on day 0, followed by immunization with different formulations containing neopeptides of ADPGK (2.5 mg/kg) on days 6, 13, and 20 posttumor inoculation. Tumor growth curves (**a**) and survival curves (**b**) were subsequently monitored (*n*  = 5). The data in (**a**) are presented as the mean ± s.d. *n*  = 5 mice per group. Survival analysis in (**b**) was performed via the log-rank test (Mantel‒Cox). **c** In a separate cohort of C57BL/6 mice receiving three consecutive immunizations with different neoantigen formulations, splenocytes were collected on day 7 after the final immunization for ELISPOT analysis of IFNγ-positive T cells to quantify neoantigen-specific T-cell responses (*n* = 5). **d**–**h** Toxicological evaluation. C57BL/6 mice received three consecutive subcutaneous injections of OVA PCD once a week. Blood samples were collected on days 7 and 14 after the final immunization for measurement of serum inflammatory cytokine levels (**d**, *n* = 5) and relevant biochemical parameters (**e**–**h**, *n* = 3), respectively. All data are expressed as mean ± s.d. Unpaired two-tailed Student’s *t* test was used for (**d–h**), and one-way ANOVA with Tukey’s post hoc test was performed for (**a**, **c**). Significance levels are indicated as **p < 0.01,***p < 0.001, ****p < 0.0001, and ns (not significant)

To validate the broad applicability of synthetic PCD vaccines, we employed a weakly immunogenic neoantigen epitope peptide (neoantigen epitope of ADPGK) derived from MC38 tumors to formulate a PCD vaccine. C57BL/6 mice were subcutaneously inoculated with MC-38 tumor cells on day 0, followed by immunization with different formulations containing neopeptides of ADPGK (2.5 mg/kg) on days 6, 13, and 20 posttumor inoculation. This enabled the assessment of the antitumor efficacy of the ADPGK PCD vaccine in MC38 tumor-bearing mice compared with that of conventional adjuvants such as CpG ODN and alum. Our results (Fig. 5a, b) demonstrated that, compared with conventional alum adjuvant or CpG ODN adjuvant, the PCD vaccine self-assembled from the neoantigen epitope peptide not only exhibited the most potent efficacy in suppressing MC38 tumor growth but also significantly prolonged the survival of tumor-bearing mice.

Furthermore, ELISPOT assays of spleens from another batch of immunized mice (Fig. 5c) further demonstrated that ADPGK PCD, compared with other traditional immune adjuvants (e.g., alum nanoparticle adjuvants and CpG ODN adjuvants), elicited a more robust tumor antigen-specific T-cell immune response characterized by higher levels of IFN-γ production, thereby enhancing antitumor immunity. These findings collectively indicate that the PCD platform possesses excellent versatility, effectively incorporating diverse antigens and enhancing their immunogenicity.

Next, we further evaluated the biosafety of PCD in vivo. The mice were immunized with OVA PCD (1 mg/kg) three times at one-week intervals. Serum samples were then collected on days 7 and 14 after the final immunization. Cytokine levels and relevant biochemical parameters were quantified. The results demonstrated that on day 7 postimmunization, the serum cytokine levels were largely within the normal range. Although the level of IL-1β slightly increased, this increase was still within the normal range (Fig. 5d), indicating that PCD did not induce significant immunotoxicity. Moreover, on day 14 postimmunization, serum biochemical parameters—including liver function markers (ALT, AST, DBIL, and TBIL) and renal function markers (creatinine (CRE) and urea nitrogen (BUN))—were not significantly different from those of the control group (Fig. 5e–h and Supplementary Fig. 13). Additionally, histopathological examination of H&E-stained sections from major organs revealed no apparent pathological alterations (Supplementary Fig. 14). Collectively, these results indicate that PCD does not cause significant physiological dysfunction or tissue damage. This study systematically validated the in vivo biosafety and tolerability profile of this PCD system, providing critical experimental evidence supporting its translational potential for further clinical applications.

### Mechanism research

**Fig. 6:**
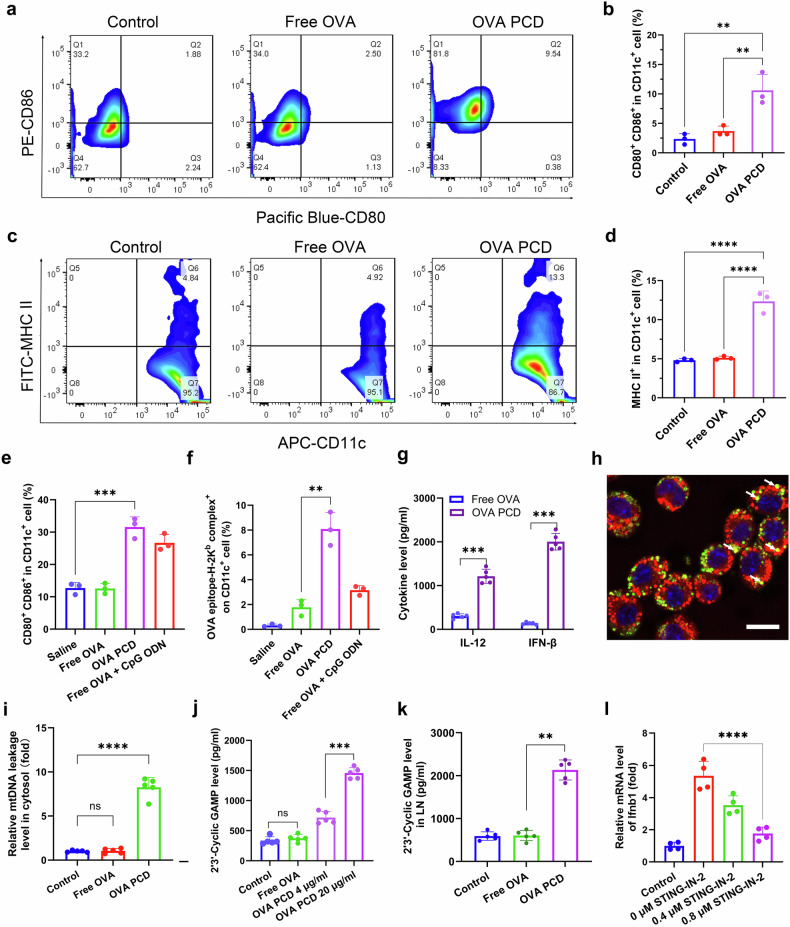
Mechanistic research. Representative flow cytometry results (**a**) and quantitative analysis (**b**, *n* = 3) of BMDC maturation. BMDCs were incubated with OVA PCD (10 μg/mL) for 48 h, followed by flow cytometry analysis of DC maturation (CD11c^+^CD80^+^CD86^+^). Representative flow cytometry plots (**c**) and quantitative analysis (**d**, *n* = 3) of MHC II molecule upregulation in BMDCs. BMDCs were incubated with OVA PCD (10 μg/mL) for 48 h, followed by flow cytometry detection of MHC II expression. Quantitative analysis of DC maturation (**e**, *n* = 3) and OVA epitope presentation (**f**, *n* = 3) in lymph nodes. C57BL/6 mice were immunized with two doses (containing an equivalent of 2.5 mg/kg OVA and 0.5 mg/kg CpG ODN). Lymph nodes were harvested 48 h after the final immunization, and single-cell suspensions were prepared for flow cytometry analysis of DC maturation and SIINFEKL-H-2Kb complex presentation on DC surfaces. **g** Cytokine levels (IL-12 and IFN-β) in supernatants measured by ELISA after BMDCs were incubated with OVA PCD (20 μg/mL) for 24 h (*n* = 5). **h** Representative fluorescence images of mitochondrial colocalization with OVA PCD. Red: mitochondria. Green: FITC-OVA PCD. Scale bar: 15 μm. After 72 h of incubation of OVA PCD with DC2.4 cells, the mitochondria were labeled with a red tracker and observed via confocal microscopy. The white arrows indicate yellow fluorescent signals from the colocalization of FITC-OVA PCD (green) with mitochondria (red). **i** qPCR analysis of mtDNA leakage in the cytosol after BMDCs were incubated with OVA PCD (20 μg/mL) for 24 h (*n* = 5). **j** cGAMP levels in BMDCs. After treatment with different concentrations of OVA PCD, ELISA was used to detect changes in the cGAMP content in BMDCs (*n* = 5). **k** cGAMP levels in the lymph nodes. C57BL/6 mice (*n* = 5) were immunized once a week 2 times. Lymph nodes were harvested 48 h after the final immunization, and single-cell suspensions were prepared for ELISA detection of cGAMP content in lymph node tissues. **l** Relative mRNA expression levels of type I interferons analyzed by RT‒qPCR after BMDCs were pretreated with different concentrations of the STING-IN-2 inhibitor followed by incubation with OVA PCD (20 μg/mL) for 24 h (*n* = 4). All data are expressed as mean ± s.d. from three independent experiments. Unpaired two-tailed Student’s *t* tests were used for (**g**), and all other statistical analyses were performed via one-way ANOVA with Tukey’s post hoc test. Significance levels are indicated as **p < 0.01, ***p < 0.001, ****p < 0.0001, and ns (not significant)

To elucidate the molecular mechanism by which the synthetic PCD vaccine activates dendritic cells (DCs), bone marrow-derived DCs (BMDCs) were coincubated with OVA PCD for 48 h. Flow cytometry analysis revealed significant upregulation of CD80/CD86 and MHC II molecules in the OVA PCD-treated group. Specifically, the percentages of CD80⁺CD86⁺ cells and MHC II⁺ cells were 10.57 ± 1.58% and 12.3 ± 0.57%, respectively, which were significantly greater than those in the free OVA group (3.72 ± 0.50% and 5.14 ± 0.11%, respectively) (Fig. 6a–d). These findings demonstrate that OVA PCD effectively promotes BMDC maturation, enhances the expression of costimulatory molecules and MHC II, and significantly improves the antigen presentation efficiency of DCs, thereby increasing their ability to activate T cells. Further flow cytometry analysis of mouse lymph nodes revealed that OVA PDC treatment significantly increased the percentage of CD80⁺CD86⁺ DCs (31.6 ± 1.9%) and the percentage of the OVA-specific epitope SIINFEKL-H-2Kb (8.07 ± 0.55%) on DCs, surpassing the percentages induced by free OVA + CpG ODN (26.7 ± 1.48%) and (3.17 ± 0.15%) (Fig. 6e, f).

Consistently, PCD treatment induced high-level secretion of IL-12 and IFN-β by BMDCs (Fig. 6g), suggesting that PCD particles alone can act as adjuvants to activate the cGAS-STING signaling pathway. Intriguingly, we observed partial mitochondrial localization of the PCD nanovaccine (Fig. 6h), indicating that PCD particles that escape lysosomes may subsequently associate with mitochondria. This was accompanied by increased mitochondrial DNA (mtDNA) leakage into the cytosol (Fig. 6i). We hypothesize that Tween 80 and sodium myristate within the nanoparticles, which act as amphiphilic surfactants, may increase mitochondrial membrane permeability and promote mtDNA release. In support of the activation of the cGAS‒STING axis, significantly elevated levels of the key second messenger cGAMP were detected in both PCD-treated BMDCs in vitro (Fig. 6j) and in the lymph nodes of treated mice (Fig. 6k). Critically, pretreatment of BMDCs with a covalent STING inhibitor significantly attenuated PCD-induced type I interferon mRNA expression (Fig. 6l), confirming the essential role of STING signaling.

To address the activation status of STING signaling in antigen-presenting cells (APCs) within the lymph nodes (LNs) of vaccinated mice, we performed the following experiment: Mice were immunized subcutaneously (s.c.) with PCD (1 mg/kg) once. Forty-eight hours after immunization, the draining lymph nodes were harvested. Single-cell suspensions of LNs were prepared, and CD11c-positive dendritic cell (DC) populations were isolated via fluorescence-activated cell sorting (FACS). Type I interferon (IFN) secretion levels and mtDNA leakage within these LN-derived DCs were subsequently assessed. As shown in Supplementary Fig. 15a, b, DCs isolated from PCD-immunized mice presented significantly enhanced features indicative of STING pathway activation compared with DCs from mice immunized with free OVA. This activation was evidenced by elevated mRNA expression of type I IFN (Supplementary Fig. 15a) and increased secretion of type I IFN (Supplementary Fig. 15b). These findings demonstrate STING activation in DCs within the lymph nodes following PCD vaccination. Consistent with the STING activation observed in LN-derived dendritic cells (DCs), as shown in Supplementary Fig. 15c, DCs isolated from the lymph nodes of PCD-immunized mice presented significantly elevated levels of mtDNA leakage compared with equivalent numbers of DCs from the control groups.

To directly assess the functional contribution of type I IFN signaling to the antitumor efficacy of the PCD vaccine, tumor-bearing mice receiving the vaccine regimen were treated with anti-mouse IFNAR-1 (IFN alpha/beta receptor subunit 1) neutralizing antibodies (clone MAR1-5A3). Subcutaneous administration of the MAR1-5A3 antibody (400 µg per dose, three doses) partially attenuated the antitumor efficacy of the PCD vaccine (Supplementary Fig. 16). The results (Supplementary Fig. 16) indicate that the PCD vaccine activates the cGAS‒STING pathway within DCs, leading to IFN-β production, which significantly contributes to the generation of a robust antitumor immune response. However, the partial nature of the reversal effect (i.e., incomplete ablation of PCD vaccine efficacy) suggests that additional mechanisms of the cGAS‒STING cascades are operative. These effects likely include STING-dependent DC maturation and the induction of other inflammatory cascades, which collectively promote the effective priming and activation of CD8^+^ cytotoxic T lymphocyte (CTL) responses.

**Fig. 7:**
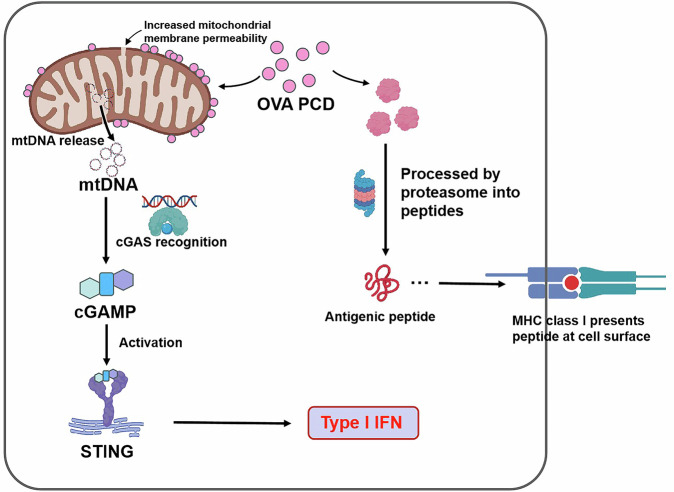
Schematic illustration of the mechanism of DC maturation induced by synthetic PCD: the PCD vaccine is partially associated with mitochondria, altering mitochondrial membrane permeability and inducing the release of mitochondrial DNA (mtDNA) into the cytosol. The released mtDNA is recognized by the cytoplasmic enzyme cyclic GMP-AMP synthase (cGAS), leading to the production of cyclic GMP-AMP (cGAMP). cGAMP then activates stimulator of interferon genes (STING), which triggers a signaling cascade. This cascade ultimately promotes the release of type I interferon (IFN-I), thereby initiating the body’s immune response and DC maturation. This schematic was created by drawing tools from MedPeer (https://www.medpeer.cn)

On the basis of these findings, we propose the following mechanism (Fig. 7): (1) lysosomal escape enables the binding of partial PCD nanoparticles to mitochondria; (2) amphiphilic surfactants in PCDs on mitochondria further destabilize mitochondrial membranes, inducing limited mtDNA leakage into the cytosol; (3) cytosolic mtDNA activates cGAS to generate cGAMP, triggering STING-dependent type I interferon production; and (4) inflammatory cytokine release and cGAS-STING pathway activation ultimately promote DC maturation and antigen-presenting function.

## Discussion

We developed a novel protein self-assembly platform utilizing amphiphilic molecules (sodium myristate/SMA and sodium dodecyl thiolate/SDT) as “molecular bridges” to form stable synthetic protein condensates (e.g., OVA-PCD) via noncovalent interactions. Driven by hydrophobic forces and disulfide bond crosslinking, this assembly strategy ensures high antigen encapsulation efficiency. Crucially, PCD nanovaccines exhibit exceptional stability and possess an intrinsic ability to evade lysosomal degradation, facilitating cytosolic antigen delivery. Mechanistically, sodium myristate (SMA) and sodium dodecanethiolate (SDT) adsorb onto protein surfaces via hydrophobic interactions. The oxidation of SDT-derived thiols (-SH) promotes intermolecular disulfide bond (-S-S-) formation, acting as molecular bridges between protein–surfactant complexes. Under weakly acidic conditions (pH 5.0), partial protonation of amphiphilic molecules enhances hydrophobicity, driving self-assembly of protein-SMA-SDT intermediates into nanoscale aggregates. Furthermore, OVA-PCD demonstrates excellent biocompatibility with minimal cytotoxicity, supporting its suitability for therapeutic applications. The protein condensate (PCD) technology developed in this study achieves aqueous self-assembly of proteins through supramolecular interactions, offering multiple advantages over conventional delivery systems. First, the synthetic PCD avoids the use of massive organic solvents (e.g., dichloromethane in PLGA preparation), thereby potentially preserving the native conformation and bioactivity of proteins, which may be critical for maintaining antigen immunogenicity. Second, leveraging a noncovalent self-assembly strategy mediated by amphiphilic molecules (sodium myristate/sodium dodecanethiolate), PCD achieves a high encapsulation efficiency of 80% and supports co-loading of multiple antigens without requiring chemical modifications or genetic engineering. These performances might surpass liposomes and polymeric nanoparticles. The synthetic PCD platform elicits robust and synergistic humoral and cellular immune responses. Immunization with OVA-PCD resulted in significantly elevated antigen-specific antibody titers, alongside potent T cell activation. This was evidenced by markedly increased frequencies of IFN-γ-producing cells and antigen-specific cytotoxic CD8⁺ T cells in the spleen. Critically, this coordinated immune activation translates into potent antitumor efficacy in vivo. In the B16-OVA melanoma model, OVA-PCD achieved significant tumor growth inhibition (up to 88%) compared to controls.

The amphiphilic components of PCD disrupt lysosomal membrane stability (Supplementary Figs. 5-6) and induce mitochondrial DNA leakage (Fig. 6h, i), synergistically activating the cGAS-STING pathway (Fig. 6j–l). This mechanism overcomes the shortcomings common to traditional carriers (e.g., PLGA and mesoporous silica nanoparticles) and significantly induces DC activation. Furthermore, the modular design of PCD ensures broad applicability, streamlined manufacturing, and high batch-to-batch consistency, highlighting its strong clinical translation potential. Compared with protein‒polymer conjugates, which require complex synthesis or genetically engineered self-assembly systems, this technology provides an efficient, safe, and scalable solution for protein self-assembly. Notably, the mechanism of cGAS-STING pathway activation—triggered by transient PCD nanoparticle interactions with mitochondria leading to the modulation of mitochondrial membrane permeability and the subsequent release of trace amounts of mtDNA—has potential implications for unintended biological effects. At excessively high concentrations, PCD nanoparticles might exhibit off-target effects, potentially causing irreversible mitochondrial damage within APCs. This damage might, in turn, precipitate cell death pathways such as apoptosis, pyroptosis, or immunogenic cell death (ICD). Therefore, despite the favorable biocompatibility profile of the condensate vaccines, maintaining the administered dose within a safe and effective therapeutic window remains a critical factor for eliciting an appropriate immune response. Moreover, this mechanistic insight reveals that low-level, stochastic mitochondrial perturbation might serve as a fundamental trigger for innate immune surveillance. In other words, the controlled release of minute quantities of pyroptotic or apoptotic signals might represent a beneficial biological process within the host, potentially acting as a key signal for sustaining innate immune defenses. This intriguing concept warrants further investigation.

PCD nanoparticles exhibit a negative zeta potential and are composed primarily of proteins and fatty acids, which results in poor cellular permeability. Theoretically, the most likely mechanism of cellular internalization involves endocytosis by APCs. Additionally, we indeed observed that upon subcutaneous administration, the PCD vaccine was preferentially trafficked to and accumulated within the lymph nodes via lymphatic drainage (Fig. 3d–f). This distribution pattern is likely attributable to the nanoparticle size facilitating retention within the lymph node architecture (Supplementary Fig. 7), thereby promoting uptake by lymph node-resident dendritic cells (DCs) (Fig. 6f).

In addition to the OVA model, the synthetic condensate vaccine platform demonstrated broad applicability, effectively enhancing the immunogenicity of a weakly immunogenic neoantigen and outperforming conventional adjuvants (alum hydroxide, CpG ODN) in suppressing tumor growth and prolonging survival in an MC38 model. This versatility, combined with a simple, scalable manufacturing process and favorable safety profile, positions PCD as a promising and adaptive strategy for next-generation vaccine development, particularly for cancer immunotherapy and potentially infectious diseases, by effectively balancing potent humoral and cellular immunity. Moreover, the ability of these condensates to target lymph nodes and stimulate strong CD8^+^ T cell-mediated immune responses addresses a critical need in cancer immunotherapy. The induction of robust cellular immune responses is essential for the effective clearance of tumors, and our findings demonstrate that these condensates can significantly enhance the antitumor efficacy of vaccines. Besides, the modular design of OVA-PCD allows for seamless integration of diverse antigens, including neoantigens and viral epitopes, positioning it as a universal platform for personalized cancer vaccines and infectious disease therapeutics. This adaptability underscores its potential to address a wide range of clinical challenges. Beyond oncological applications, this new nanoadjuvant system shows broad therapeutic potential against microbial pathogens (viruses, Mycobacterium tuberculosis, mycoplasma), highlighting its clinical translation prospects.

To further facilitate the clinical translation of PCD technology, the following studies are required. Scalable manufacturing processes should be optimized to ensure product stability and batch-to-batch consistency at production scale, and long-term toxicity in large-animal models should be examined to support early-phase clinical evaluation. The molecular mechanisms underlying PCD-mediated lysosomal escape should be further elucidated by integrative approaches—including molecular-dynamics simulations, and high-resolution structural imaging (e.g., cryo-EM)—so that a mechanistic basis for the rational design of cytosolic-delivery platforms can be established. The application scope of PCD may be broadened; for example, its use as a vehicle for genome-editing or for targeted delivery needs to be explored. Personalized PCD-based vaccines may be developed by combining PCD with specific antibodies to enable precise delivery. Collectively, these studies will deepen mechanistic insight into PCDs, expand its biomedical applications, and accelerate its development as an innovative therapeutic modality.

## Materials and methods

### Materials and reagents

Ovalbumin (OVA, ≥98%), sodium hydroxide (99%), sodium myristate, Cy3/5.5-NHS, dithiothreitol (DTT), Tween-20, and Tween-80 were purchased from Aladdin. Dodecyl thiol was obtained from Sigma‒Aldrich. LyssoTracker Green was obtained from Thermo Fisher. The SIINFEKL (OVA_257-264_) peptide was synthesized by Shanghai Bioengineering Co., Ltd. CpG ODN and the Alhydrogel® adjuvant were purchased from InvivoGen. HA protein (catalog number: 11085-V08H) and SARS-CoV-2 S glycoprotein (catalog number: 40589-V08H26) were purchased from Sinobiological. The ADPGK mutant peptide (ASMTNMELM) was custom synthesized by Sangon Biotech (Shanghai) and was >95% pure, as verified by HPLC‒MS analysis. The Bradford protein assay kit was purchased from Shanghai Bioengineering Co., Ltd. The CCK-8 assay kit was obtained from Abbkine. TMB substrate solution was purchased from Macklin. 5-(N-ethyl-N-isopropyl) amiloride (EIPA), chlorpromazine (CPZ) and genistein were purchased from MCE. The E-TOXATE™ Kit was purchased from Sigma‒Aldrich. The ELISPOT kit was obtained from Diaclone. Brefeldin A solution (1000×) was purchased from BioLegend (catalog no. 420601). FcX™ (anti-mouse CD16/32; 101319; BioLegend), anti-mouse CD80-PE (Catalog No. 104708; BioLegend), APC anti-mouse CD11c (117310; BioLegend), Pacific Blue™ anti-mouse CD80 (104723; BioLegend), PE anti-mouse CD86 Antibody (105007; BioLegend), APC anti-mouse CD3 Antibody (100235; BioLegend), FITC anti-mouse CD8a Antibody (100705; BioLegend), PE anti-mouse IFN-γ (505807; BioLegend), Pacific Blue™ anti-mouse CD3 (100213; BioLegend), PE anti-mouse H-2Kb bound to SIINFEKL Antibody (141603, BioLegend), FITC anti-mouse I-A/I-E (107605, BioLegend), Zombie Aqua™ Fixable Viability Kit (423101, BioLegend), 7-AAD viability staining solution (420403, BioLegend), OVA antibody (B441199, BioLegend), and HRP-conjugated goat anti-mouse (STP249, Seyotin) were used. T-Select MHC tetramer (H-2K^+b^-restricted SIINFEKL; TS-5001-1C; MBL) and HRP-goat anti-mouse IgG (A21010; Abbkine) were used. All other chemical reagents were of analytical grade and were obtained through commercial channels. DC2.4, MC38, RAW 264.7 and 293 T cells were obtained from ATCC. The B16-OVA-GFP and B16-OVA cells are stable expression cell lines constructed via lentiviral transduction to introduce the exogenous OVA gene into B16 cells.

### Preparation and characterization of OVA protein condensates (PCDs)

OVA was mixed with sodium myristate (SMA) at a molar ratio of 1:400 and stirred for 20 min. Dodecyl thiol and sodium hydroxide were dissolved in anhydrous ethanol in equimolar amounts to react and form sodium dodecane-1-thiolate (0.1 mol/L, SDT). SDT was then mixed with the OVA-SMA mixture at various molar ratios (OVA:SDT = 1:50, 1:100, 1:200, 1:300) and stirred for an additional 30 min to form OVA condensates. The OVA-SMA-SDT mixture was transferred into a dialysis bag with a molecular weight cutoff of 50 kDa and dialyzed overnight in PBS (pH = 5.0) to remove free components, yielding purified OVA condensates (OVA PCD). The dialyzed OVA PCD solution was combined with Tween 80 (0.5 mg/mL) and thoroughly mixed. The mixture was subsequently centrifuged at 12,000 rpm for 20 min to isolate the particulate fraction. The collected particles were lyophilized and stored at −20 °C until further use. Sterile pure water was used for rehydration in subsequent experiments. The protein content was determined via the traditional Bradford method. The protein encapsulation efficiency (EE%) was calculated by measuring the OVA content in the OVA PCD particles resuspended after centrifugation. The formula for calculating protein encapsulation efficiency was EE% = (mass of protein actually encapsulated/total mass of protein initially added) × 100%. The morphology of OVA PCD was examined via transmission electron microscopy (TEM) via negative staining with a 2% phosphotungstic acid solution before imaging, and the particle size, zeta potential, and size stability were monitored via dynamic light scattering (DLS). The endotoxin content was quantified via the Limulus amebocyte lysate (LAL) assay according to the manufacturer’s instructions. ADPGK PCD and HA-SARS-CoV-2 S PCD were prepared via identical fabrication procedures.

### Cell culture

MC38, RAW 264.7 and 293 T cells were cultured in DMEM (Gibco) supplemented with 10% fetal bovine serum (FBS) and 1% penicillin/streptomycin at 37 °C in a 5% (v/v) CO_2_ atmosphere. B16-OVA and DC2.4 cells were cultured under the same conditions in RPMI-1640 medium (Gibco) supplemented with 10% FBS and 1% penicillin/streptomycin.

### Bone marrow-derived dendritic cell (BMDC) culture

Bone marrow cells were isolated from the femurs and tibiae of C57BL/6 mice. These cells were cultured in complete medium consisting of RPMI-1640 medium supplemented with 10% (vol/vol) heat-inactivated fetal bovine serum (FBS), 50 U/mL penicillin/streptomycin, 1 mM sodium pyruvate, 25 mM HEPES, 55 µM β-mercaptoethanol, and 20 ng/mL recombinant murine GM-CSF (PeproTech). Fresh complete medium containing GM-CSF was replenished on day 3. On day 6, nonadherent cells were collected from the culture supernatant, and loosely adherent cells were harvested by gentle washing with PBS. These pooled cells were used for subsequent experiments.

### Isolation and culture of mouse splenocytes

In brief, the mice were euthanized at the experimental endpoint, and their spleens were aseptically harvested. The spleens were ground to collect splenocytes, and red blood cells were thoroughly removed via red blood cell lysis buffer. The isolated splenocytes were cultured in RPMI-1640 medium (Gibco) containing 10% FBS and 1% penicillin/streptomycin at 37 °C in a 5% (v/v) CO_2_ atmosphere.

### Flow nano analyzer analysis

The FITC-labeled HA protein and Cy3-conjugated SARS-CoV-2 spike (S) glycoprotein were coassembled via the established fabrication protocol, followed by single-particle fluorescence profiling of the resulting PCDs via ultrasensitive nanoflow cytometry (NanoFCM).

### In vitro release study

To determine the release characteristics of OVA PCD in PBS (pH 7.4), 1 mL of purified OVA PCD solution (OVA concentration of 1.33 mg/mL) was sealed in a dialysis bag (MW cutoff of 50 kDa) and placed in 10 mL of PBS (pH 7.4). The solution contained in the dialysis bag was shaken at 70 rpm at 4 °C. At 12, 24, 48, 72, 96, 120, and 144 h, 100 µl of PBS outside the dialysis bag was taken out and replaced with fresh PBS to maintain the volume. The OVA content in the PBS at each time point was determined via the Bradford method. To simulate in vitro release under reducing conditions, 20 mM dithiothreitol (DTT) was added to OVA PCD, and the cumulative release of free OVA was measured at 0.5 h, 1 h, 3 h, and 8 h.

### Fluorescence recovery after photobleaching (FRAP) assay

A mixture of sodium myristate (SMA) and FITC-labeled ovalbumin (FITC-OVA) was observed via confocal microscopy. A defined region of interest (ROI) within the particles was photobleached via a 488 nm laser at 5% intensity for 0.5 s, followed by continuous monitoring of fluorescence recovery kinetics.

### In vitro cytotoxicity assay

The in vitro cytotoxicity of OVA PCD was evaluated via a CCK-8 assay. 293 T cells were seeded into 96-well plates at a density of 5 × 10^3^ cells per well and cultured for 24 h. Different concentrations of OVA PCD (10 µg/mL, 30 µg/mL, 50 µg/mL, 80 µg/mL, or 100 µg/mL) were added, and the mixture was incubated with the 293 T cells for 24 h. After the medium containing OVA PCD was removed, 100 µL of complete medium containing 10 µL of CCK-8 solution was added to each well, and the mixture was incubated for 30 min. The absorbance was measured at 450 nm. The relative cell viability was calculated via the following formula: Cell viability (%) = (At - Ab)/(Ac - Ab) × 100%, where At, Ab, and Ac represent the absorbances of treated cells, blank (no cells), and untreated cells, respectively. In addition, a CTL cytotoxicity assay was used to evaluate the cytotoxic effect of effector T cells from the spleens of mice after antitumor immunotherapy on tumor cells in vitro.

### In vitro cellular uptake and lysosomal escape assay

RAW 264.7 or BMDC cells were cultured in confocal dishes at a density of 1 × 10^5^ cells. The cells were then incubated with Cy3-NHS-labeled OVA PCD (2.5 μg/mL) for 12, 24, 48, or 72 h, followed by staining with LysoTracker Green (50 nM) and Hoechst 33342 (5 μg/mL). After each treatment, the cells were washed three times with PBS and observed via a laser scanning confocal microscope (LSM 880). DC2.4 cells were incubated with OVA PCD (5 μg/mL) for 12, 24, 48, or 72 h to assess cellular uptake and lysosomal escape kinetics following the established protocol.

For the cellular uptake mechanism experiment, DC2.4 cells were pretreated for 4 h with the following uptake inhibitors: chlorpromazine (CPZ, 10 μM) to block clathrin-mediated endocytosis, genistein (Gen, 200 μM) to inhibit caveolae-mediated endocytosis, and 5-(N-ethyl-N-isopropyl)amiloride (EIPA, 1 mM) to inhibit macropinocytosis. The DC2.4 cells were subsequently incubated with FITC-labeled OVA PCD for 12 h and then analyzed via flow cytometry.

### In vivo imaging of OVA PCD

For the lymph node drainage studies, OVA PCD and free OVA were prelabeled with the near-infrared fluorescent dye Cy5.5. C57BL/6 mice were treated with Cy5.5-OVA PCD or Cy5.5-OVA via subcutaneous injection at the tail base. Under isoflurane anesthesia, in vivo near-infrared fluorescence imaging was performed via a multimodal small animal imaging system (FX Pro) at 12 and 24 h post-injection. At the corresponding time points, the mice were euthanized, and the inguinal lymph nodes were harvested for ex vivo fluorescence signal acquisition.

### OVA PCD-enhanced humoral immune response study

To investigate the humoral immune response to OVA PCD, BALB/c mice were subjected to two subcutaneous immunizations with OVA PCD, OVA + CpG ODN (CpG ODN administered at 0.5 mg/kg), or free OVA, separated by 2 weeks. The primary immunization consisted of 50 μg of OVA, and the booster immunization consisted of 25 μg. Fourteen days post-booster, blood was collected via retro-orbital sinus puncture. After 30 min of incubation at room temperature, the serum was obtained by centrifugation at 3000 rpm for 10 min at 4 °C and stored at −80 °C. ELISA was used to measure total IgG and IgG subclass levels in the serum, with the results expressed as the OD450.

### Immunohistochemistry and ELISA

The mice were injected subcutaneously at the tail base with 50 μg of OVA PCD or free OVA. After 24 h, the lymph nodes were harvested, fixed in 4% formaldehyde for 12 h, and processed for paraffin embedding and sectioning. The sections were deparaffinized and hydrated, followed by antigen retrieval and blocking with endogenous peroxidase. Nonspecific binding was blocked with 3% serum. The sections were incubated with primary antibodies overnight at 4 °C and then with secondary antibodies. DAB was used to visualize positive signals, and the staining intensity was monitored microscopically. Nuclei were counterstained with hematoxylin, and the sections were dehydrated, cleared, and mounted. Blue-stained nuclei and brown–yellow-positive cells were observed and analyzed under a microscope. In addition, bone marrow-derived dendritic cells (BMDCs) or lymph node-derived dendritic cells (DCs) from immunized mice were plated at a density of 1 × 10^5^ cells per well and cultured for an additional 24 h. The concentration of IFN-β in the cell culture supernatant was then quantified via an enzyme-linked immunosorbent assay (ELISA) kit (Invitrogen, 424001).

### In vivo antitumor study of OVA PCD

C57BL/6 mice (6–8 weeks old, 18–20 g) were subcutaneously injected at the tail base with OVA PCD or free OVA on days 0, 7, and 21. The initial injection dose was 50 μg OVA equivalent, while the subsequent doses were halved to 25 μg OVA equivalent. The control group received an equal volume of saline. On day 28, seven days after the final injection, the mice were subcutaneously inoculated with 5 × 10^5^ B16-OVA tumor cells in the right thigh. Tumor growth was monitored for 18 consecutive days, and mouse body weight and tumor volume were recorded every two days. The tumor volumes were calculated via the following formula: (length × width^2^)/2. On day 18, the experiment was terminated, and the mice were euthanized. Subcutaneous tumors were excised, weighed, and photographed. Additionally, the spleens were aseptically harvested and ground, and the splenocytes were collected for flow cytometry analysis.

### In vivo antitumor study of ADPGK PCD

C57BL/6 mice (6‒8 weeks old, 18‒20 g) were inoculated with 2 × 10^5^ MC-38 tumor cells (s.c.). On days 6, 13, and 20 post-tumor inoculation, immunizations were subsequently performed via different formulations containing ADPGK neopeptide (2.5 mg/kg) (*n* = 5). Tumor growth and survival rates were monitored. In another batch of C57BL/6 mice (*n* = 5) that received three consecutive immunizations with different ADPGK neoantigen formulations, splenocytes were collected on day 7 after the final immunization for ELISPOT analysis of IFNγ-positive T cells.

### Dendritic cell maturation

Splenocytes were washed with PBS and resuspended in FACS buffer (1 × 10⁶ cells/100 μL). The cells were blocked with anti-CD16/32 for 30 min at RT, followed by surface staining with fluorochrome-conjugated antibodies against CD11c, CD80, and CD86 for 20 min at 4 °C in the dark. Then, viability was assessed via the use of 7-AAD.

### IFN-γ⁺ CD8⁺ T-cell detection

Splenocytes (1 × 10^6^ cells/well) were stimulated with SIINFEKL peptide (8 μg/mL) and brefeldin A in complete medium for 6 h at 37 °C and 5% CO₂. The cells were washed, stained with a fixable viability dye (Aqua™), and blocked with anti-CD16/32. Surface staining was performed with anti-CD3 and anti-CD8 antibodies for 30 min at 4 °C. The cells were fixed (4% PFA), permeabilized (0.1% Triton X-100), and stained intracellularly with anti-IFN-γ antibodies. The cells were washed, and images were acquired after filtration.

### Tetramer staining

Splenocytes (1 × 10^6^ cells/well) were pulsed with the SIINFEKL peptide (8 μg/mL) for 72 h. The cells were washed, stained with viability dye (Aqua™), blocked with anti-CD16/32, and stained with the PE-conjugated H-2Kb/SIINFEKL tetramer for 30 min at 4 °C. Surface staining was performed with anti-CD8 antibodies prior to flow cytometry analysis. Unless otherwise specified, the viability of splenic and lymph node-derived immune cells was assessed via a fixable viability dye (Aqua™). Dendritic cell maturation analysis was performed with 7-AAD for viability staining.

### ELISPOT assay

The experiment was conducted according to the kit instructions. Briefly, the ELISPOT plates were pretreated with 35% ethanol and washed five times with sterile water. The plates were then coated with capture antibody at 4 °C overnight, and unbound antibodies were washed away with sterile water. Freshly isolated splenocytes were transferred to ELISPOT plates, and 8 μg/mL SIINFEKL was added to each well for a 24-h incubation. After incubation, the cells were washed away, and the plates were incubated with the detection antibody at room temperature for 2 h. The plates were then washed with sterile water and incubated with a streptavidin‒AP conjugate at room temperature for 1 h, followed by five washes with PBS. Finally, BCIP/NBT substrate solution was added to the plates, which were subsequently incubated at room temperature until approximately 10–15 min. The chromogenic reaction was terminated by multiple washes with sterile water. After drying naturally, the spots were counted via an IRIS ELISpot/Fluorospot reader from Mabtech.

### mtDNA isolation and qPCR analysis

After coculture of BMDCs with OVA PCD (20 μg/mL), cytoplasmic mtDNA leakage was analyzed by qPCR. Using a commercial Beyotime mitochondrial isolation kit, the cells were fractionated into nuclear, mitochondrial, and cytoplasmic compartments. Specifically, cells were homogenized under cold conditions and subjected to low-speed centrifugation to remove nuclei and intact mitochondria, followed by high-speed centrifugation to collect the supernatant (cytoplasmic fraction). DNA was then purified from the cytoplasmic fraction via a DNA extraction kit, and mitochondrial-specific (e.g., mt-CO1) and nuclear (e.g., 18S rDNA) gene fragments were amplified via qPCR. The relative copy numbers of cytoplasmic mtDNA and nuclear DNA were compared to objectively evaluate mtDNA cytoplasmic translocation.

### 2’3’-cGAMP level analysis

ELISA (Invitrogen) was performed to measure the 2’3’-cGAMP content in BMDCs after treatment with different concentrations of OVA PCD (4 μg/mL and 20 μg/mL). C57BL/6 mice (*n* = 5) were immunized weekly for two doses. Lymph nodes were harvested 48 h after the final immunization, and single-cell suspensions were prepared for ELISA quantification of cGAMP levels in lymph node tissues.

### Toxicological evaluation

Healthy mice with similar body weights were randomly divided into two groups (*n* = 5). The experimental group received subcutaneous injections of 1 mg/kg OVA PCD at the tail base, whereas the control group received equivalent volumes of saline. All three injections were administered once a week at the same time point. Blood samples were collected on day 7 after the final injection for cytokine detection. On day 14, blood samples were again collected for biochemical analysis, and cardiac, hepatic, splenic, pulmonary, and renal tissues were harvested for histopathological sectioning and hematoxylin and eosin (H&E) staining.

### Mice

Female C57BL/6 mice and BALB/c mice (6–8 weeks old) were purchased from the Guangdong Experimental Animal Center. The mice were housed in rooms maintained at a temperature of 22 °C to 25 °C with a 12-h light/12-h dark cycle. Food and water were provided ad libitum. This study adhered to relevant ethical regulations for animal testing and research. All animal experiments were approved by the Ethics Committee of the Medical Laboratory Animal Center of Southern Medical University (SMUL202404047).

### In vivo IFNAR blockade

C57BL/6 mice were subcutaneously inoculated with 8 × 10^5^ B16-OVA cells on day 0, followed by subcutaneous administration of the PCD vaccine + anti-IFNAR neutralizing antibody (catalog #BE0241; BioXCell) on days 6, 11, and 16. Tumor growth and survival were monitored throughout the study. The anti-IFNAR antibody was administered at 400 μg per mouse per dose, while the PCD vaccine was given at 1 mg/kg per immunization. The control groups (saline, OVA PCD alone, and anti-IFNAR antibody alone) were treated in parallel via identical protocols.

### Statistical analysis

Statistical analyses were performed via GraphPad Prism 9. Unpaired two-tailed Student’s *t* tests were used to evaluate significant differences between two groups. One-way or two-way analysis of variance (ANOVA) was used for comparisons among multiple groups. For statistically significant intergroup differences, post hoc pairwise comparisons were performed via Tukey’s test. A *p* value of less than 0.05 was considered statistically significant. All significant values shown in various figures are indicated as follows: *P < 0.05, **P < 0.01, ***P < 0.001 and ****P < 0.0001.

## Supplementary information

Supplementary information

## Data Availability

All data supporting the findings of this study are available within the article and its supplementary information.
