# Supplementary material for: Condensate nanovaccine adjuvants augment CD8+ T-Cell-dependent antitumor immunity through mtDNA leakage-triggered cGAS-STING axis activation
Source: Signal Transduct Target Ther. 2025 Oct 21;10:349. doi: 10.1038/s41392-025-02447-w (PMC12537970; doi:10.1038/s41392-025-02447-w)
Supplement: Supplementary file 1 — Supplementary information [file 41392_2025_2447_MOESM1_ESM.docx]

Supplementary materials for

**Condensate nnovaccine adjuvants augment CD8^+^ T-Cell-dependent antitumor immunity through mtDNA leakage-triggered cGAS-STING axis activation**

*Yu Tang^1, †^, Zhiyuan Luo ^2, †^, Zhanni Ma^1, †^, Lingling Han^1, †^,* *Yurong Zhou^3, †^, Tianci Liang^1^, Kangsen Yang^1^, Lei Zhao^4^, Xiaoyuan Chen^5, 6, 7*^, Pengfei Zhang^1*^*

Correspondence to: [zpf0418@i.smu.edu.cn](mailto:zpf0418@i.smu.edu.cn(P.Z.);) or chen.shawn@nus.edu.sg (X.C.)

**This PDF file includes:**

Figures. S1 to S16


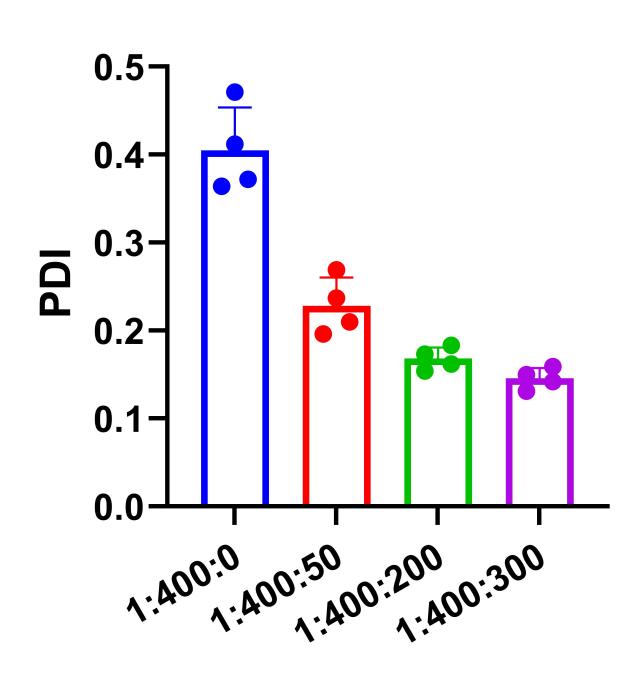


**Figure. S1.** Detection of PDI for PCDs formed at different assembly ratios (protein:SMA:SDT) by DLS. Data are presented as the mean ± s.d. (n = 4) from three independent experiments.


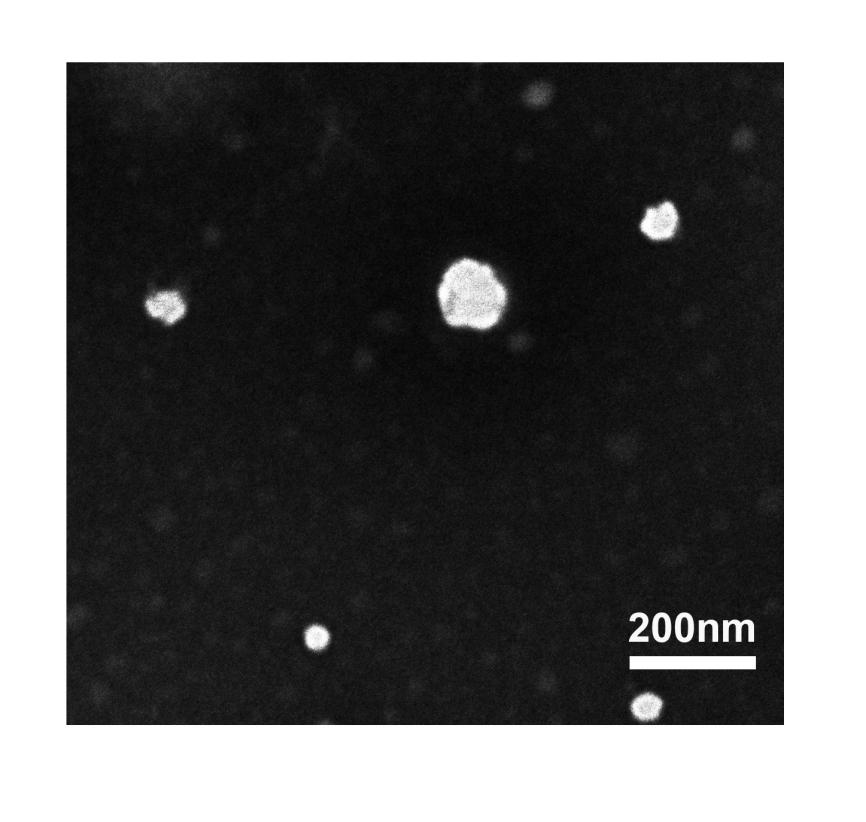


**Figure. S2.** Transmission electron microscopy (TEM) analysis of OVA PCD after lyophilization and reconstitution shows that OVA PCD maintains intact spherical structure post lyophilization and reconstitution, with particle size distribution of 50-100 nm, demonstrating no significant changes compared to pre-lyophilization. Scale bar: 200 nm.


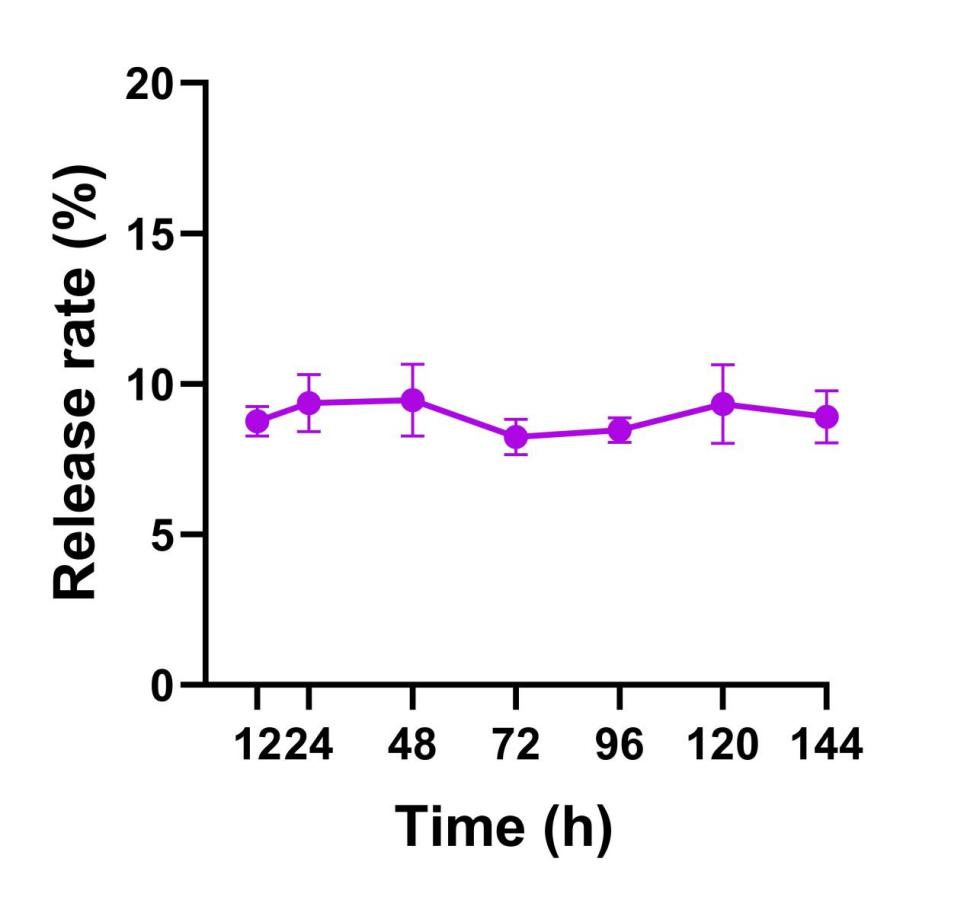


**Figure. S3.** In vitro release profile of OVA PCD under simulated physiological conditions (pH 7.0 PBS). The cumulative release rate reached approximately 9% within 144 hours, confirming the in vitro stability of OVA PCD. Data are presented as mean ± s.d. (n = 3) from three independent experiments.


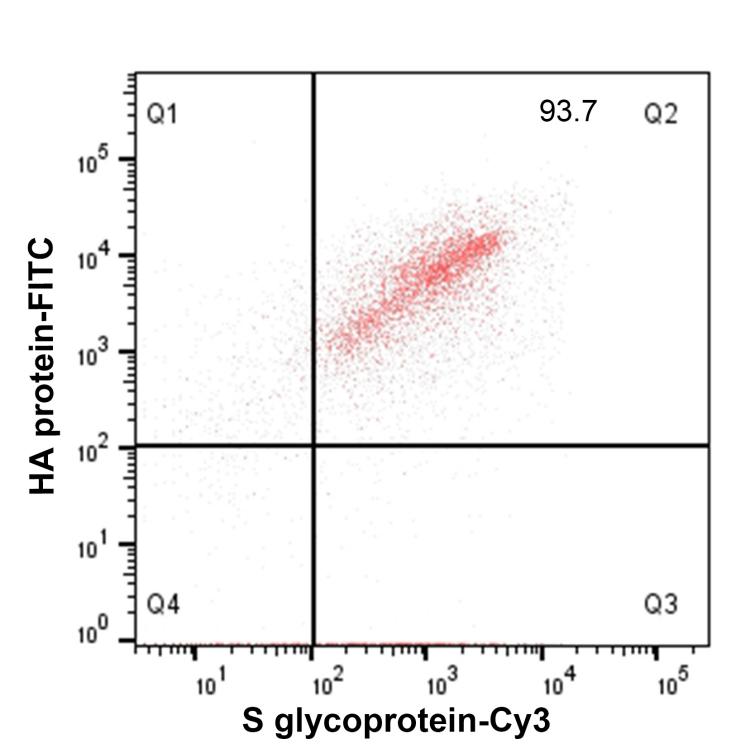


**Figure. S4.** Using a dual-fluorescence labeling strategy, FITC-labeled HA protein and Cy3-labeled SARS-CoV-2 S glycoprotein were co-assembled, followed by single-particle fluorescence analysis of the co-assembled PCD using ultrasensitive nano-flow cytometry (Nano FCM). Nano FCM detection results showed that the co-assembly efficiency of the two viral surface proteins reached 93.7%, demonstrating that this assembly method can simultaneously incorporate two different protein antigens to form condensate vaccine nanoparticles.


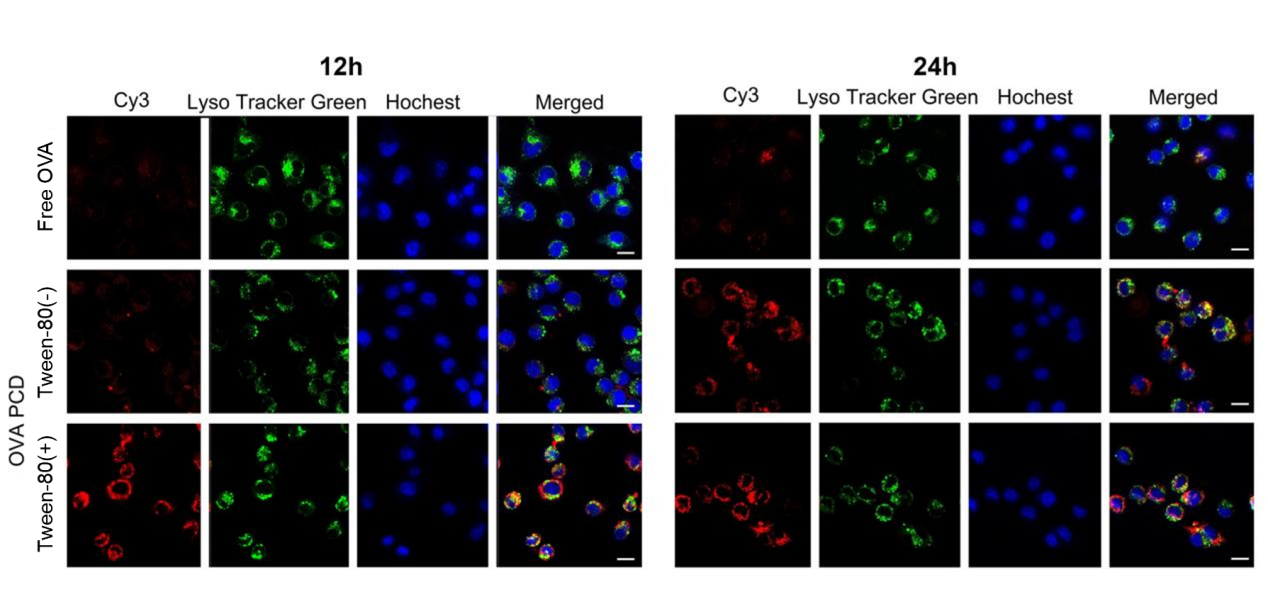


**Figure. S5.** Representative fluorescence confocal microscopy images showing lysosomal colocalization analysis of DC2.4 cells incubated with synthesized PCD (5 μg/mL) for 12 h and 24 h. The results demonstrate time-dependent cellular uptake characteristics of OVA PCD, with Tween 80 significantly enhancing its uptake efficiency. Red: Cy3-OVA PCD. Blue: Cell nuclei. Scale bar: 10 μm.


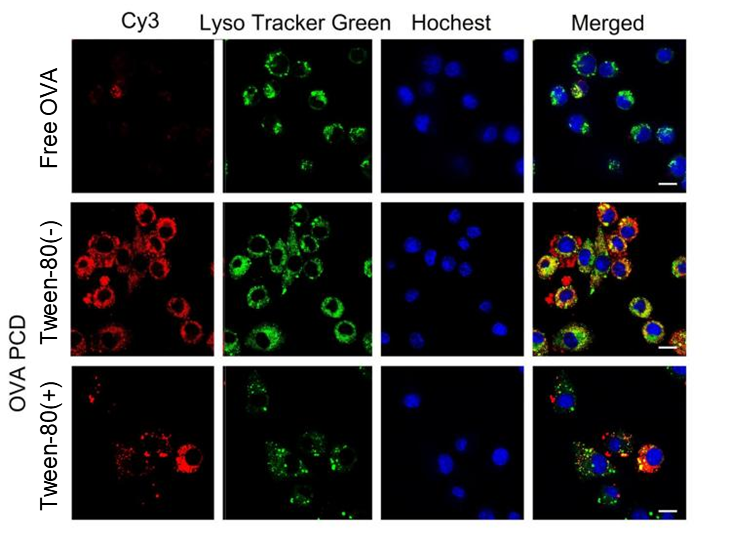


**Figure. S6.** Representative fluorescence confocal microscopy images of lysosomal colocalization analysis for the synthetic PCD (5 μg/mL) after 48 h co-incubation with DC2.4 cells. Red: Cy3-OVA PCD. Green: Lysosomes. Blue: Cell nuclei. Scale bar: 10 μm.


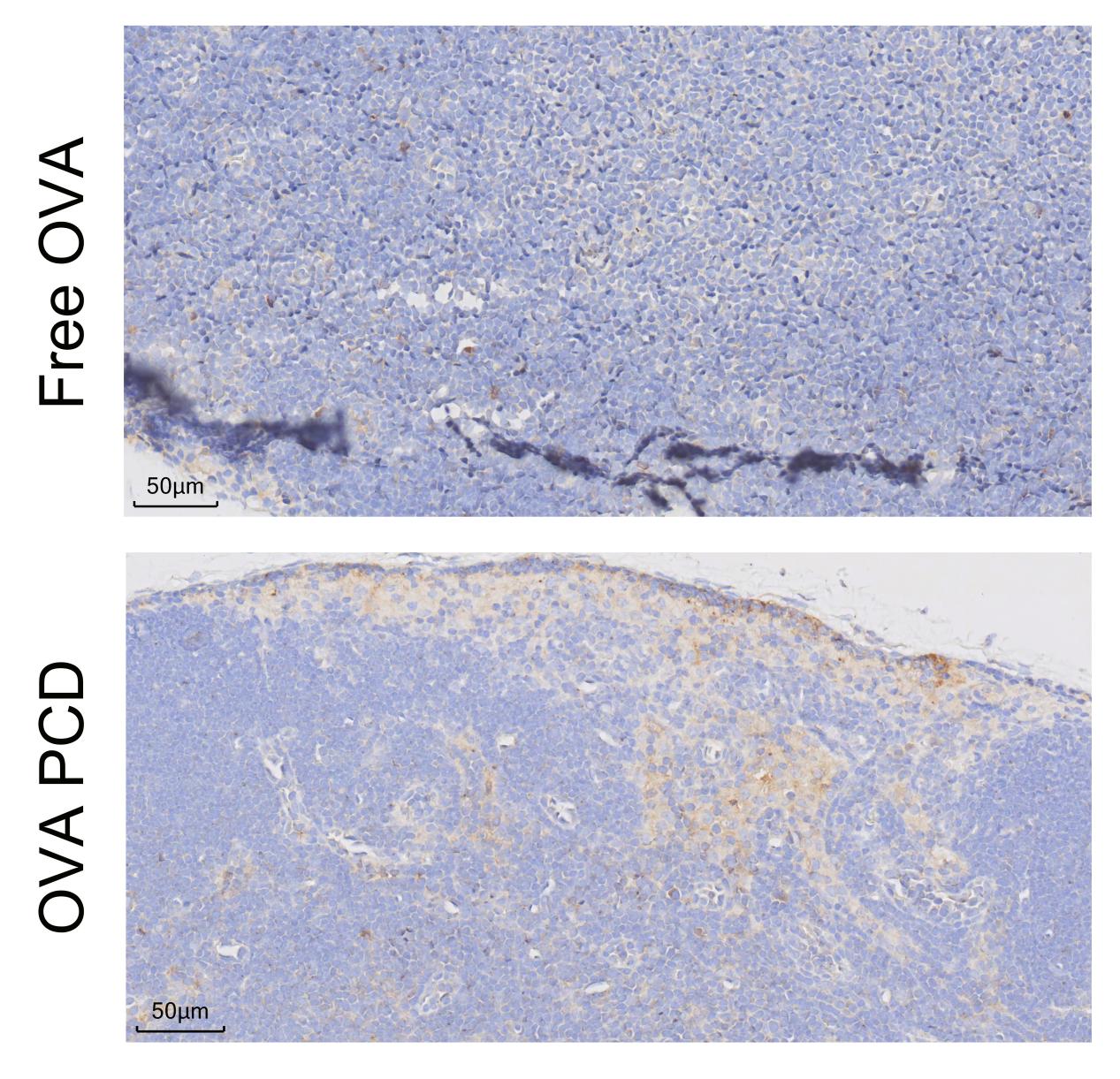


**Figure. S7.** Immunohistochemical staining analysis of passive drainage targeting to draining lymph nodes following subcutaneous injection of OVA PCD at the tail base. Brown: Positive signal. Blue: Cell nuclei. Scale bar: 50 μm.


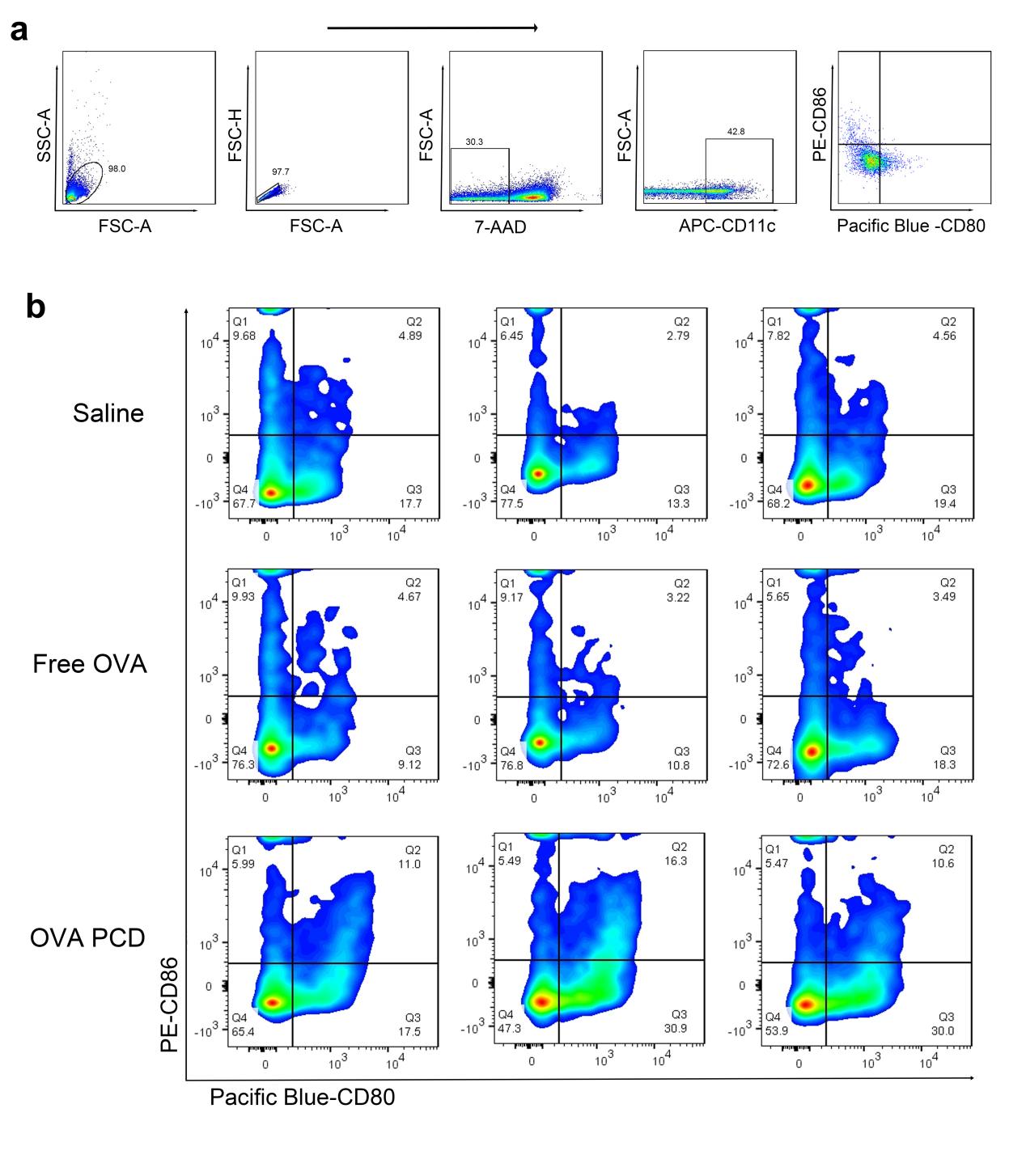


**Figure. S8.** **a** Gating strategy for flow cytometric analysis of DC maturation. Singlet cell populations were identified using forward scatter area vs height parameters (FSC-A vs FSC-H), viable cells were selected, and CD8^+^ T cells were further gated for functional analysis. **b** Flow cytometry plots showing mature DCs (CD80^+^ CD86^+^ CD11C^+^) in splenic samples from mice post-OVA PCD antitumor treatment.


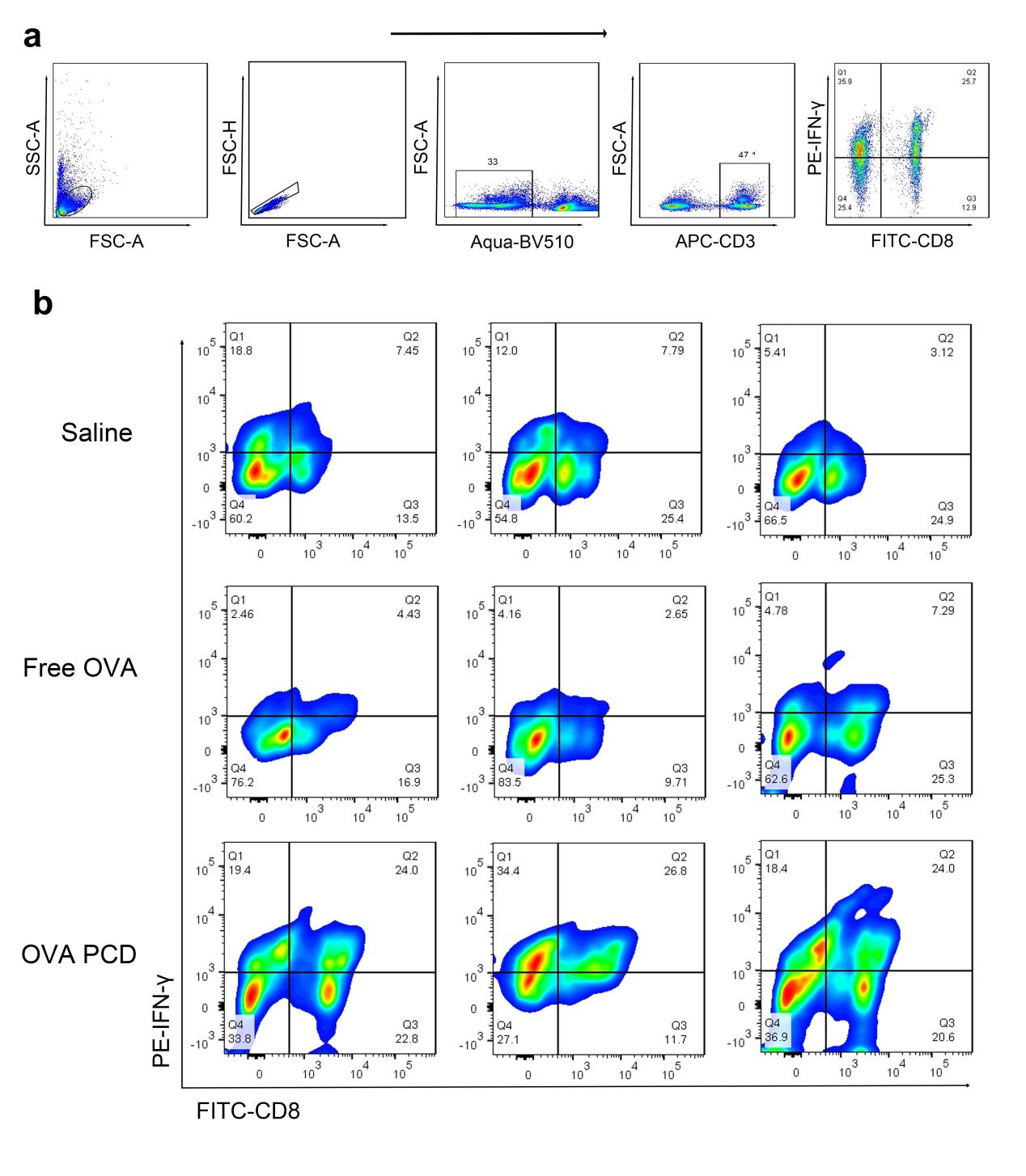


**Figure. S9.** **a** Gating strategy for IFN-γ flow cytometry. Singlet cells were determined by FSC-A vs FSC-H, viable cells were selected, and CD8^+^ T cells were gated for analysis. **b** Flow cytometry plots of IFN-γ^+^ subsets among splenic CD8^+^ T cells post-OVA PCD antitumor treatment.

**
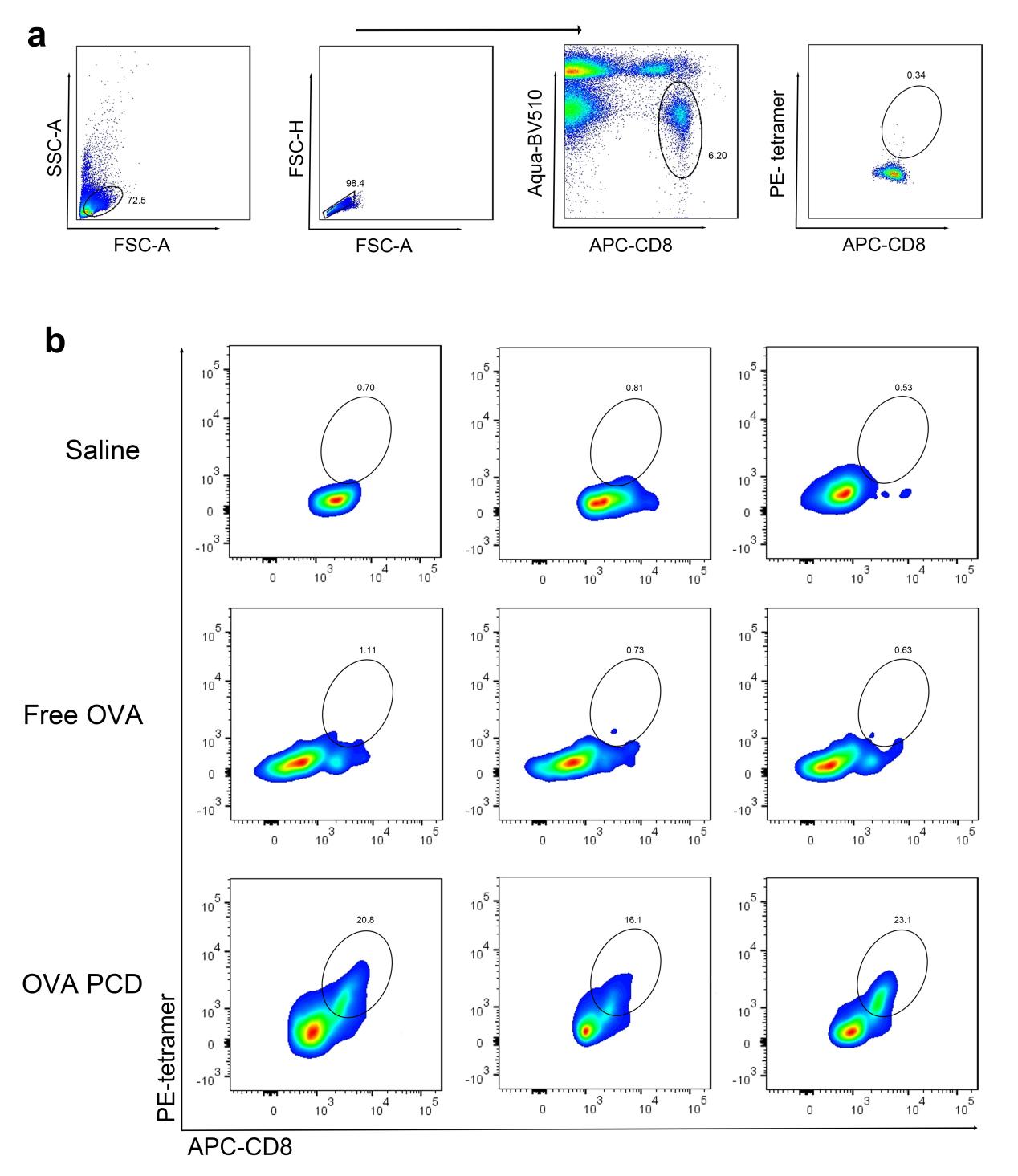
Figure. S10. a** Gating strategy for OVA tetramer flow cytometric analysis. Singlet cell populations were confirmed via FSC-A vs FSC-H, viable cells were selected, and CD8^+^ T cells were gated for further analysis. **b** Flow cytometry plots of OVA-specific tetramer-positive CD8^+^ T cell subsets in splenocytes after OVA PCD antitumor therapy.


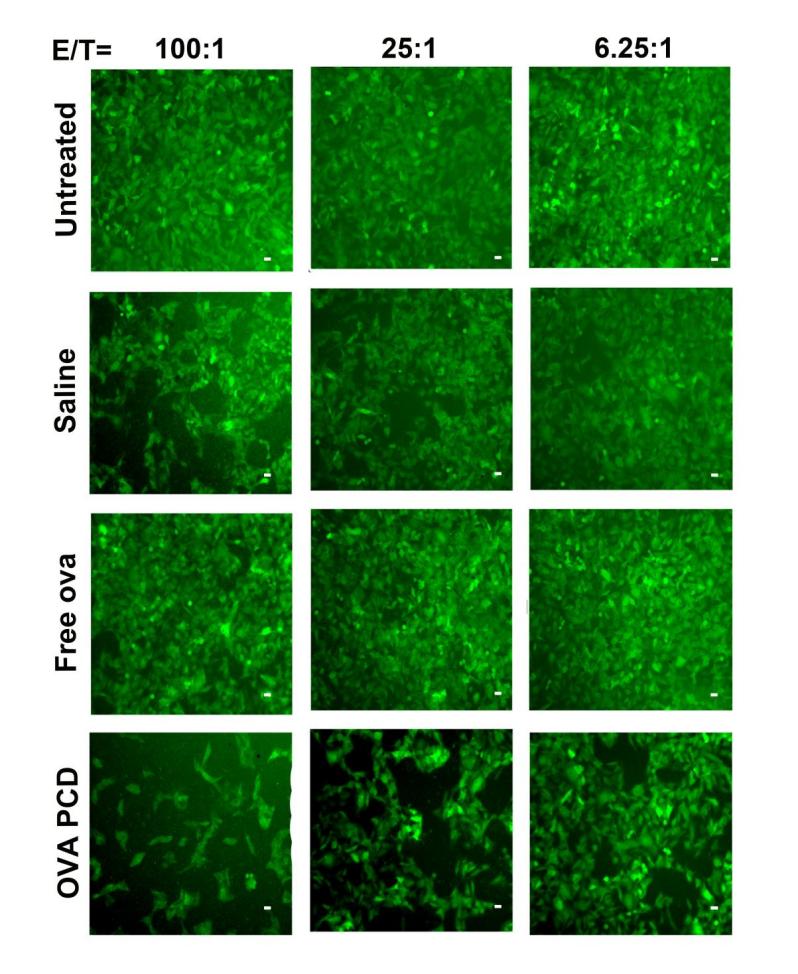


**Figure. S11.** Representative fluorescence microscopy images of effector splenocytes (E) co-incubated with target B16-OVA-GFP cells (T) at specified ratios for 24 hours in vitro. Scale bar: 10 μm.


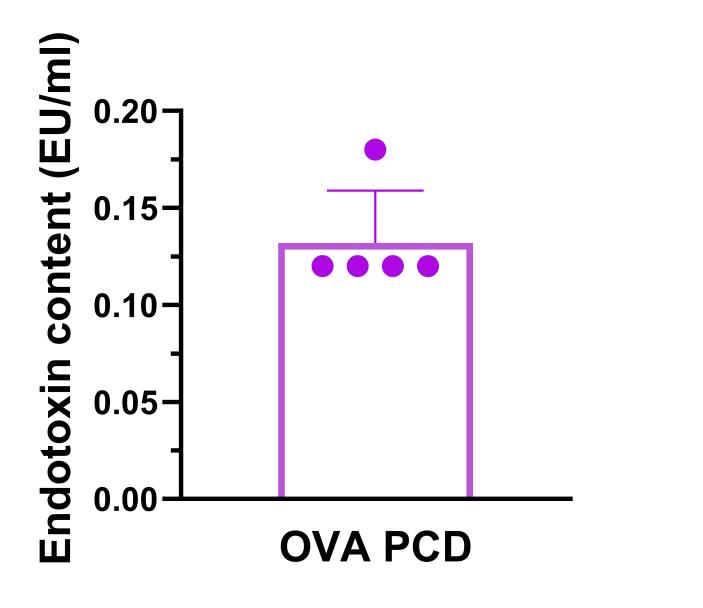


**Figure. S12.** Endotoxin quantification in OVA samples, showing negligible endotoxin levels (n = 5). Data are presented as the mean ± s.d. from three independent experiments.


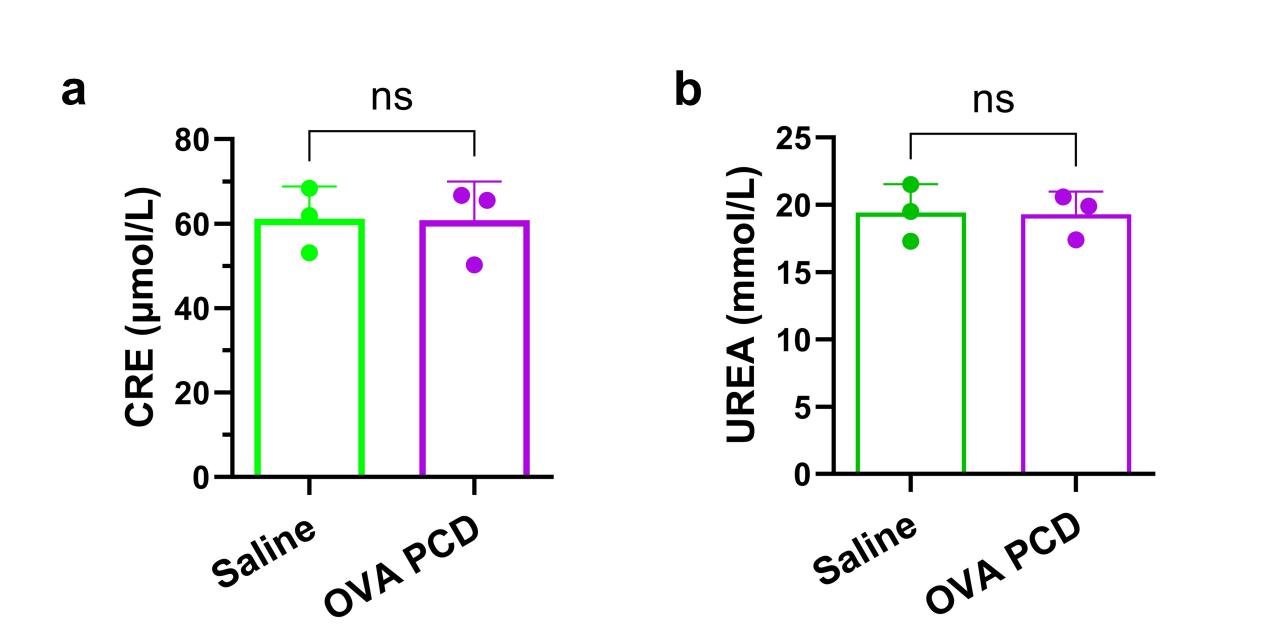


**Figure. S13.** OVA PCD bio-safety assessment: Serum creatinine (CRE, **a**) and urea (UREA, **b**) level analysis. C57BL/6 mice were administered three subcutaneous injections of OVA PCD (1 mg/kg, n = 3), once a week. Blood samples were collected on day 14 after the final immunization, and creatinine (CRE) and urea (UREA) concentrations were measured using enzymatic methods to evaluate potential renal toxicity. All data are expressed as mean ± s.d.. All data were analyzed using an unpaired two-tailed Student’s t-test. Significance levels are indicated as ns (not significant).


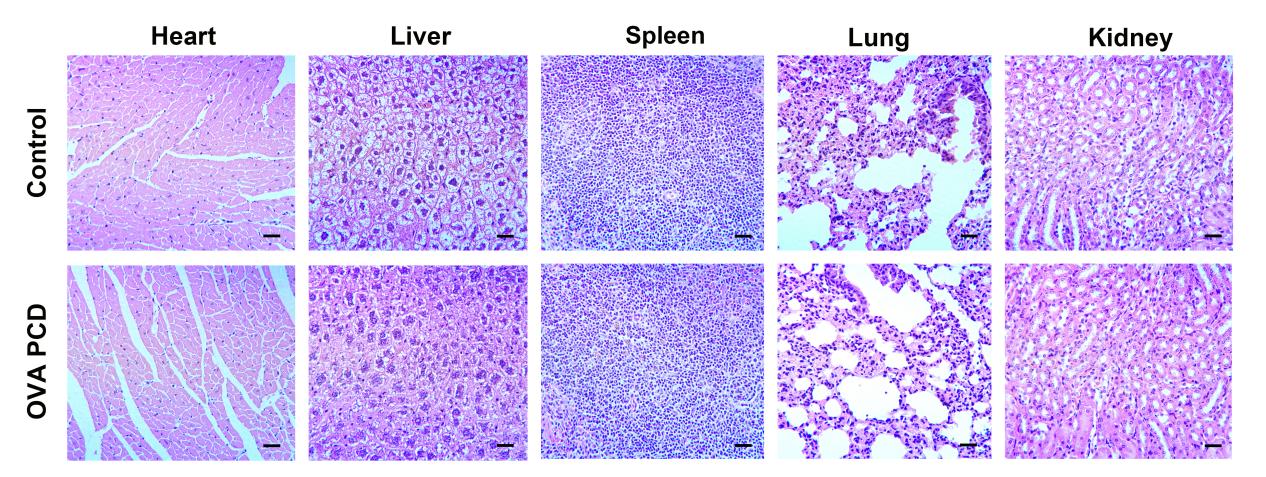


**Figure. S14.** Representative HE images of major organs (heart, liver, spleen, lungs, kidneys) from C57BL/6 mice after three subcutaneous OVA PCD injections (1 mg/kg, n=5), weekly. Tissues collected on day 14 post-final immunization were fixed and stained for histopathological assessment. Scale bar: 100 μm.


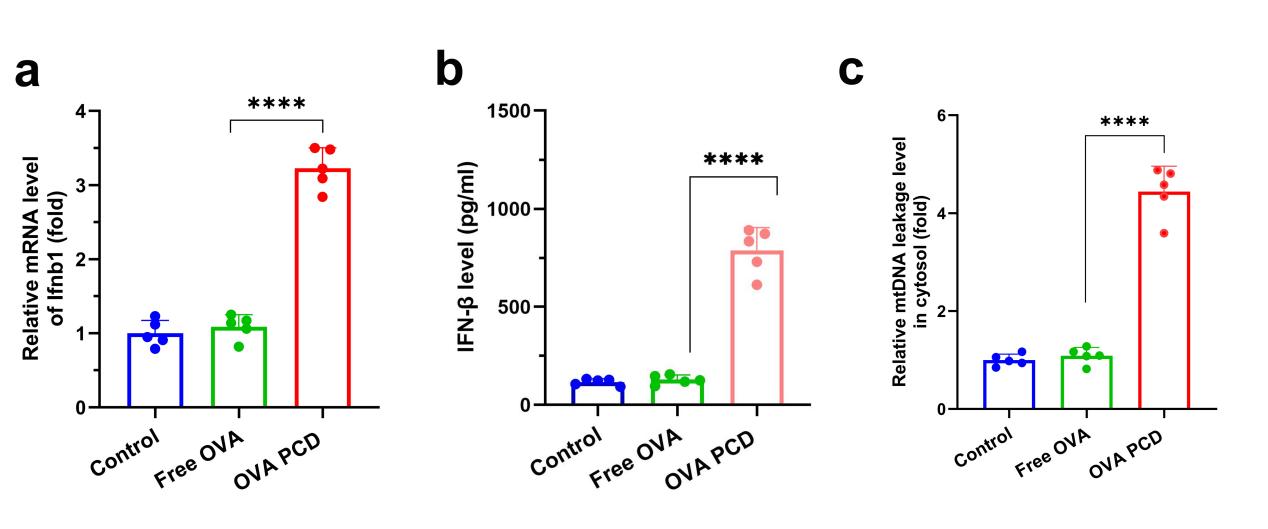


**Figure. S15.** Healthy C57BL/6 mice were subcutaneously immunized once with different formulations (1 mg/kg OVA). At 48 hours after the immunization, the lymph nodes were collected and processed into single-cell suspensions. CD11c-positive dendritic cell (DC) populations were then sorted by flow cytometry. The type I IFN levels (**a**-**b**) and mtDNA leakage (**c**) in equal numbers of DCs were detected by ELISA or qPCR. All data are expressed as mean ± s.d. from two independent experiments (n = 5). Groups were compared using one-way ANOVA with Tukey's post hoc test. Significance levels are indicated as *****p*<0.0001.


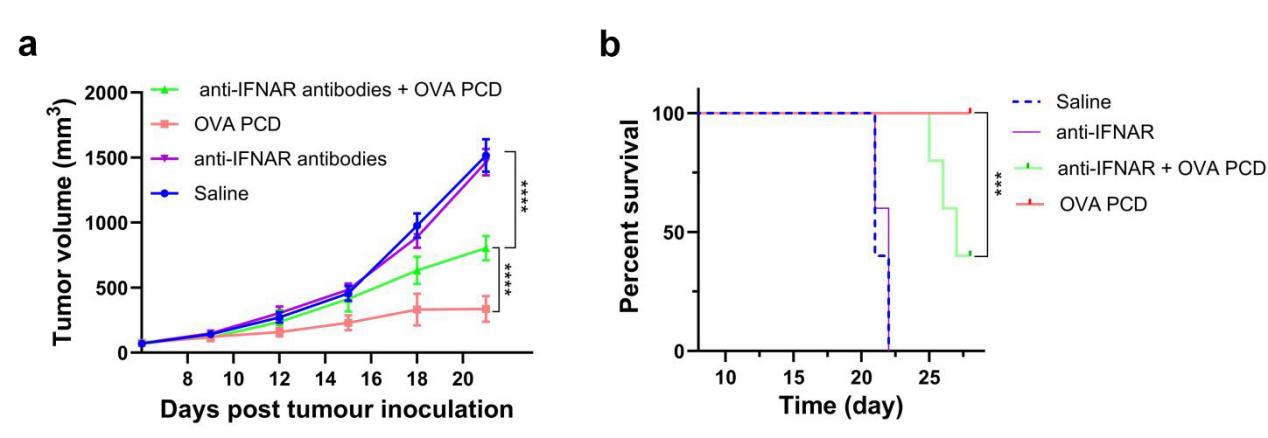


**Figure. S16.** C57BL/6 mice were subcutaneously inoculated with 8×10^5^ B16-OVA cells on day 0, followed by subcutaneous immunization with different formulations (containing either 1 mg/kg OVA or 400 μg anti-IFNAR1 antibodies per mouse) on days 6, 11, and 16. Tumor growth (**a**) and mouse survival (**b**) were then monitored. Statistical data are presented as the mean ± s.d. (n = 5) . Groups in **a** were compared using one-way ANOVA with Tukey's post hoc test. Survival analysis uses the log-rank Mantel-Cox test. Significance levels are indicated as****p*<0.001, *****p*<0.0001, and ns (not significant).
